# Constitutively active glucagon receptor (GCGR) physiologically regulates body temperature in lizards

**DOI:** 10.1126/sciadv.aeb8744

**Published:** 2026-08-21

**Authors:** Songsong Liu, Qingqing Ren, Shanshan Lai, Xiangying Xiang, Yongjie Huang, Qingqing Wang, Qiujinman Mao, Xiaofei Yan, Ao Dai, Najeeb Ullah, Hong Li, Yin Qi, Yanfu Qu, Xiang Ji, Juergen Brosius, Cheng Deng

**Affiliations:** ^1^ Jiangsu Key Laboratory for Biodiversity and Biotechnology, State Key Laboratory of Microbial Technology, College of Life Sciences, Nanjing Normal University, Nanjing, PR China.; ^2^ Department of High Altitude Medicine, Center for High Altitude Medicine, College of Life Sciences, West China Hospital, Sichuan University, Chengdu, Sichuan, PR China.; ^3^ Institute of High Altitude Medicine, High Altitude Medicine Key Laboratory of Sichuan Province, West China Hospital, Sichuan University, Chengdu, Sichuan, PR China.; ^4^ Chengdu Institute of Biology, Chinese Academy of Sciences, Chengdu, Sichuan 610299, China.; ^5^ Zhejiang Provincial Key Laboratory for Water Environment and Marine Biological Resources Protection, College of Life and Environmental Sciences, Wenzhou University, Wenzhou 325035, China.; ^6^ Center for High Altitude Medicine, Xining, Qinghai, P. R. China.

## Abstract

Endotherms use complex internal mechanisms to maintain a steady body temperature, whereas ectotherms rely on environmental factors and behaviors for thermoregulation. Certain ectothermic animals, like some lizards, can also physiologically regulate their body temperature to some extent, with interspecies variations in thermoregulatory capacity under low-temperature conditions. However, the molecular mechanisms underlying this physiological regulation have remained unclear since its discovery about 80 years ago. Here, we reveal that the hepatic expression of constitutively active glucagon receptor (GCGR) in lizards, particularly in iguana, correlated with their thermoregulatory capacity when kept at low temperatures. When *Pogona vitticeps* and *Phrynocephalus vlangalii* with thermoregulatory capacity were treated with short hairpin RNA–*GCGR* or GCGR antagonist, their body temperatures dropped, and the expression of genes related to energy metabolism and respiratory metabolic rate was down-regulated. In contrast, *Eremias argus*, lacking thermoregulatory ability, revealed low hepatic GCGR expression. Overexpression of *P. vitticeps GCGR* (pv*GCGR*) in the liver of *E. argus* increased body temperature and enhanced energy metabolism. Furthermore, in mice with reduced thermoregulatory ability by chlorpromazine administration, overexpression of constitutively active GCGR ligand-independently increased body temperature under cold conditions. In conclusion, constitutively active GCGR drives the physiological thermoregulation in lizards, and our study offered previously unidentified insights into expression regulation of constitutively active GPCR into physiological adaptations responding to environmental changes.

## INTRODUCTION

Temperature is a major environmental factor affecting many biological aspects of organisms, including physiology, morphology, geographical distribution, and behavior (*1*, *2*). All organisms respond to environmental challenges through adaptive mechanisms (*3*). Thermoregulation is an essential mechanism for maintaining body temperature stability and can be divided into behavioral and autonomic regulation (*4*). Behavioral regulation is faster and more energy efficient. For instance, animals exposed to cold or hot environments typically regulate their body temperature by adjusting their posture or moving to a different thermal environment. However, if these behaviors prove insufficient or fail to meet immediate thermal demands, autonomic responses are activated (*5*). Autonomic thermoregulation is much more refined and involves physiological mechanisms (*6*).

The physiological functions of animals are influenced not only by intrinsic constraints but also by environmental factors, such as low ambient temperatures (*7*). Endothermic and ectothermic animals use distinct physiological strategies for thermoregulation (*8*). Endotherms (e.g., mammals and birds) maintain stable internal body temperature independent of external environmental conditions (*9*).

Ectothermic organisms are more dependent on external environmental conditions for body temperature regulation (*10*). This thermal dependence has been extensively studied to understand the molecular mechanisms underlying thermoregulation in those species (*11*). The earliest study of how lizards regulate their metabolism (e.g., oxygen consumption at different environmental temperatures) were conducted by Regnault and Reiset in the 1840s (*12*). About a century later, Cowles and Bogert observed a proportional increase in metabolism with the rise of external temperature and explored the role of physiological thermogenesis in lizards (*13*). Since then, reports of physiological thermoregulation of lizards have followed, for example, Bartholomew and Tucker noted that *Pogona vitticeps* adapts to high temperatures by altering heart rate and metabolism (*14*). Other studies have highlighted species-specific differences in how lizards regulate their body temperature in response to changes in the environment (*15*). These mechanisms include altering metabolism, controlling blood flow and heart rate (*16*), and modulating hormone levels (*17*).

Notably, the fatty acid biosynthesis pathway is substantially up-regulated in certain lizards (e.g., *Eremias argus*) at low temperatures (*18*). This type of regulation enables animals to maintain a relatively constant body temperature under various environmental conditions (*17*). However, specific lizard gene pathways, which are responsible for maintaining a relatively constant body temperature at low temperatures remain unidentified.

G protein–coupled receptors (GPCRs) play essential roles in both physiological processes and diseases, making them highly sought-after targets for drug development. These receptors interact with G proteins (e.g., G_s_, G_i_, G_q_, and G12) to transmit signals from the extracellular environment to intracellular effector pathways (*19*). Among all vertebrates, type B GPCRs—including the glucagon receptor (GCGR), glucagon-like peptide-1 receptor (GLP1R), glucagon-like peptide-2 receptor (GLP2R), and gastric inhibitory peptide receptor (GIPR)—exhibit high sequence similarity and functional conservation (*20*). The GCGR, is primarily expressed in the liver of all vertebrates (*21*). The GCGR-ligand axis play a crucial role in glucose homeostasis and energy metabolism (*22*), particularly in mammals, where it modulates glucose homeostasis and energy expenditure (*23*). Upon activation, GCGR stimulates hepatic glucose production through glycogenolysis, thereby increasing blood glucose levels to maintain energy balance. Two key enzymes, phosphoenolpyruvate carboxykinase (PEPCK) and glucose-6-phosphatase (G6PC), play integral roles in the GCGR regulatory pathway (*24*). In addition, the GCG-GCGR axis directly increases the metabolism rate and energy expenditure by up-regulating fibroblast growth factor 21 (FGF21) in rodents (*25*). FGF21, a metabolic regulator primarily secreted by the liver in response to metabolic stressors, such as fasting and nutrient deprivation, modulates energy metabolism (*26*). Activation of GCGR through glucagon signaling influences FGF21 expression, forming a regulatory loop that adjusts metabolic responses to fluctuating energy demands (*27*).

Many GPCRs have been found to produce second messengers even in the absence of ligands, which is defined as constitutive activity (*28*) and is associated with various potential physiological functions (*28*–*30*). In a previous study, we reported that avian GCGR displays high constitutive activity, with activity levels correlating with blood glucose (*31*). In the present work, we uncovered that lizard GGCR displays high constitutive activity while also maintaining high expression levels in the liver, in sync with their thermoregulatory capacity. Our central hypothesis is that lizards sustain thermoregulation via high expression and constitutive activity of hepatic GCGR. Accordingly, under cold conditions, thermoregulating lizards should exhibit considerably higher GCGR expression than those lacking this ability. Thus, we compared hepatic GCGR expression in lizards with and without thermoregulation abilities under cold conditions. Using adeno-associated virus (AAV)–mediated GCGR overexpression, short hairpin RNA (shRNA)–AAV–*GCGR* knockdown, or pharmacological antagonists, we explored the role of GCGR in physiological thermoregulation at organismal and cellular levels, offering previously unknown insights into metabolic adaptations and environmental interactions in ectothermic animals. For a complete list of abbreviations and a compilation of animal species (Latin and common names) and their abbreviations, see tables S1 and S2.

## RESULTS

### Hepatic expression of GCGR in lizards at low temperatures correlates with thermoregulatory capacity

**Fig. 1.**
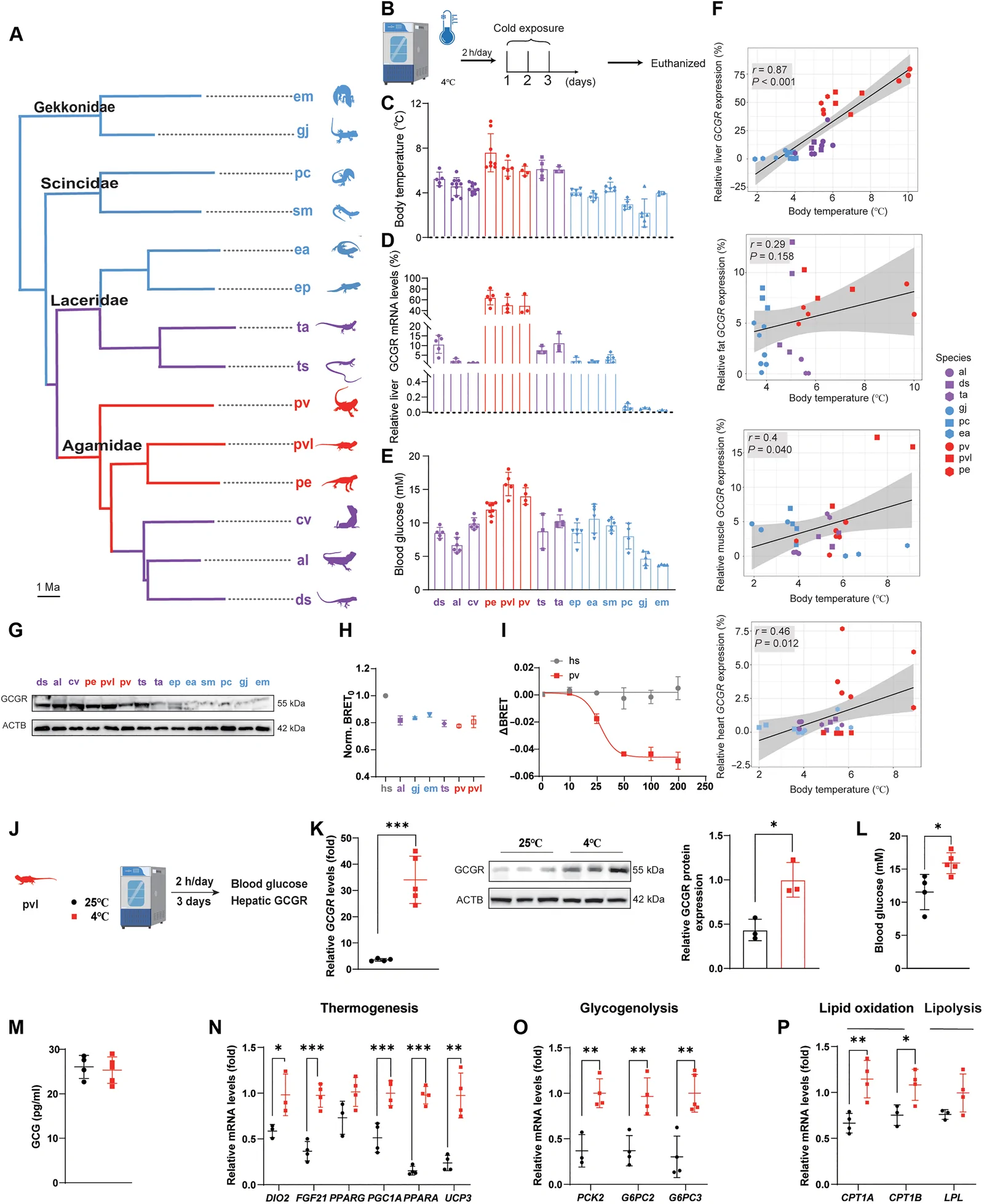
Hepatic GCGR correlates with lizard thermoregulation at 4°C. (**A**) Phylogenetic tree of lizard species. al, *Acanthosaura lepidogaster*; ds, *D. splendidum*; cv, *Calotes versicolor*; ea, *E. argus*; ep, *E. przewalskii*; em, *Eublepharis macularius*; gj, *G. japonicus*; pvl, *P. vlangalii*; pe, *P. erythrurus*; pv, *P. vitticeps*; pc, *Plestiodon chinensis*; sm, *S. modesta*; ta, *Takydromus amurensis*; ts, *Takydromus septentrionalis*; Ma, million years ago. (**B** to **E**) Lizards were exposed for 2 hours at 4°C followed by 22 hours (h) at 25°C. After the third cold-period, temperature was immediately measured, followed by decapitation and measurements of the following parameters (B): Body temperature (*n* = 3 to 10) (C) and, after euthanizing, hepatic *GCGR* mRNA expression levels (*n* = 3 to 6) (D) and blood glucose (*n* = 3 to 8) (E) were measured. Different colors represent different thermoregulatory abilities: red (high), purple (medium), and blue (relatively weak). (**F**) Correlation between GCGR mRNA and body temperature across tissues in lizards with varied thermoregulatory capacities at 4°C. (**G**) Hepatic GCGR protein levels in different lizard species after adapting to low temperatures for 3 days. (**H**) Normalized GCGR BRET_0_ values from different lizard species. (**I**) ΔBRET concentration-response curves of pv*GCGR* (ng). hs, *Homo sapiens*. (**J**) One group of *P. vlangalii* was kept constantly at 25°C for 3 days; the other group included a time period of 2 hours at 4°C. At the end of the last cold exposure, animals of both groups were euthanized and parameters were measured: (**K** to **P**) hepatic *GCGR* mRNA (left; *n* = 5) and protein (middle and right; *n* = 3) expression (K), blood glucose levels (*n* = 4 to 5) (L) and GCG levels (*n* = 4 to 5) (M), hepatic mRNA levels of thermogenesis-related genes (*PGC1A*, *UCP3*, *FGF21*, *DIO2*, *PPARA*, and *PPARG*) (N), glycogenolysis-related genes (*G6PC2*, *G6PC3*, and *PCK2*) (O), and lipid metabolism–related genes (*CPT1A*, *CPT1B*, and *LPL*) (*n* = 3 to 4) (P) of both *P. vlangalii* groups (*n* = 3 to 5). Data are represented as means ± SD and analyzed by unpaired Student’s *t* test (K to P).

We experimentally exposed lizards that we could capture or purchase to controlled temperatures gradients and measure their body temperatures and blood glucose levels. Most of the iguana species, including *Phrynocephalus vlangalii*, *P. vitticeps*, *Diploderma splendidum*, *Phrynocephalus erythrurus*, and *Phrynocephalus przewalskii* (*32*–*34*), exhibited such thermoregulatory characteristics raising their body temperatures 1° to 3°C in relation to the environmental temperature (4°C) (Fig. 1, A to C). We found that, despite similar body sizes of *P. vlangalii* and *E. argus*, the warming and cooling rates of *P. vlangalii* were higher than that of *E. argus* under the same conditions, with a relatively small thermal time constant, which was in line with our expectations (fig. S1, A to C). In addition, although *P. vitticeps* had a larger body size than both *P. vlangalii* and *E. argus*, the thermal time constant was the smallest, indicating that it had a superior thermoregulatory capacity (fig. S1, A to C, and table S3). Furthermore, there appears to be a correlation between blood glucose levels and thermoregulatory capacity (Fig. 1E and fig. S2B). Also, only in the liver, we found significant correlation between GCGR expression and body temperature at 4°C (Fig. 1, C, D, F, and G, and fig. S2, C and D). On the other hand, glucagon (GCG) levels were not different among different lizards (fig. S2A). We also found that, in thermoregulating lizards exposed to 4° and 25°C, GCGR expression exhibited a consistent thermogenesis, correlating with glycogenolysis and lipid metabolism (Fig. 1, J to P). We show that human embryonic kidney (HEK) 293T cells expressing lizards GCGR exhibited constitutive G_s_ pathway activation (Fig. 1, H and J, and fig. S2E). To verify whether these elevated expression levels contributed to physiological effects, we overexpressed mouse or lizard-derived *GCGR*s (mm*GCGR* and pv*GCGR*) in alpha mouse liver 12 cells (AML12). Overexpression of pv*GCGR* greatly up-regulated *Fgf21*, peroxisome proliferator–activated receptor gamma, coactivator 1 alpha (*Pgc1a*), peroxisome proliferator–activated receptor alpha (*Ppara*), and *G6pc1* expression (fig. S2, F and G). In contrast, we observed no significant changes in expression of these genes in mm*GCGR*-transfected cells (fig. S2, F and G).

### *P. vlangalii* lost physiological thermoregulation after knocking down hepatic *GCGR*

**Fig. 2.**
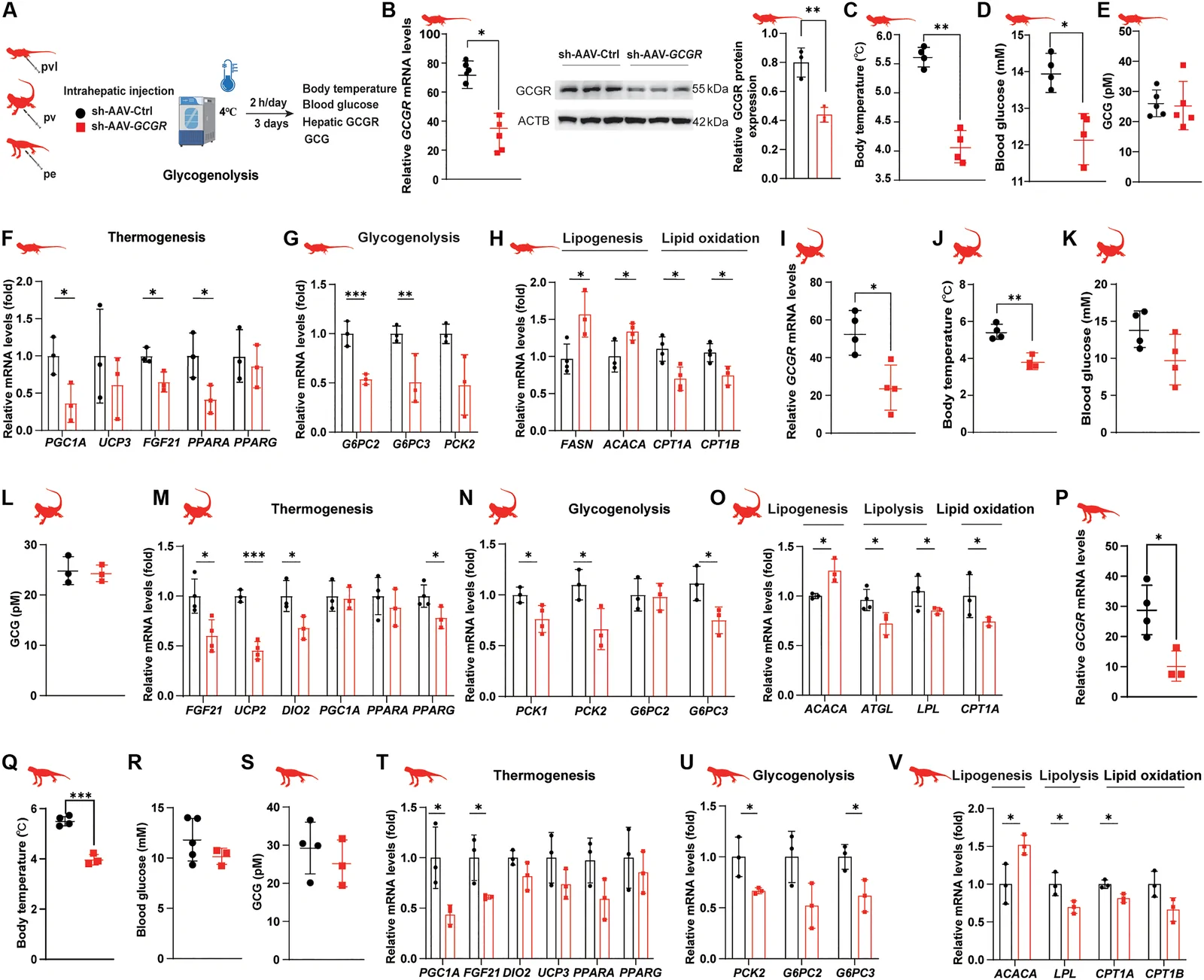
Hepatic GCGR knockdown disrupts lizard thermoregulation. (**A**) Lizards (*P. vlangalii*, *P. vitticeps*, and *P. erythrurus*) received intrahepatic injection of shRNA-AAV-Ctrl and shRNA-AAV-*GCGR* into the liver followed by 2 hours of exposure to 4°C and 22 hours at 25°C. After the third cold-period, temperature was measured, followed by decapitation and measurements of the following parameters in *P. vlangalii* liver: (**B** to **E**) Hepatic *GCGR* mRNA (left; *n* = 5) and protein (middle and right; *n* = 3) expression (B), body temperature (*n* = 4) (C), blood glucose levels (*n* = 4) (D), and GCG levels (*n* = 5) (E) of *P. vlangalii* after the third 2 hours of exposure to 4°C. Hepatic mRNA levels of thermogenesis-related genes (*PGC1A*, *UCP3*, *FGF21*, *PPARA*, and *PPARG*) (*n* = 3 to 4) (**F**), glycogenolysis-related genes (*G6PC2*, *G6PC3*, and *PCK2*) (*n* = 3 to 4) (**G**), and lipid metabolism–related genes (*FASN*, *ACACA*, *CPT1A*, and *CPT1B*) (*n* = 3 to 4) (**H**) in the *P. vlangalii* after the third two-hour exposure to 4°C. (**I** to **O**) *P. vitticeps* liver: hepatic *GCGR* mRNA expression (*n* = 4) (I), body temperature (*n* = 4) (J), blood glucose levels (*n* = 4) (K), and GCG levels (*n* = 3) (L). Hepatic mRNA levels of thermogenic genes (*PGC1A*, *DIO2*, *UCP2*, *FGF21*, *PPARA*, and *PPARG*) (*n* = 3 to 4) (M), glycogenolysis-related genes (*G6PC2*, *G6PC3*, *PCK2*, and *PCK1*) (*n* = 3 to 4) (N), and lipid metabolism–related genes (*ACACA*, *ATGL*, *LPL*, and *CPT1A*) (*n* = 3 to 4) (O). (**P** to **V**) *P*. *erythrurus* liver: hepatic *GCGR* mRNA expression (*n* = 3 to 4) (P), body temperature (*n* = 3 to 4) (Q), blood glucose levels (*n* = 3 to 4) (R), and GCG levels (*n* = 3 to 4) (S). Hepatic mRNA levels of thermogenesis-related genes (*PGC1A*, *UCP3*, *FGF21*, *PPARA*, *PPARG*, and *DIO2*) (*n* = 3) (T), glycogenolysis-related genes (*G6PC2*, *G6PC3*, and *PCK2*) (*n* = 3) (U), and lipid metabolism–related genes (*ACACA*, *LPL*, *CPT1A*, and *CPT1B*) (*n* = 3) (V). Black represents the shRNA-AAV-Ctrl group, and red represents the shRNA-AAV-*GCGR* group. *P < 0.05; **P < 0.01; ***P < 0.001. Data are represented as means ± SD, analyzed using unpaired Student’s *t* test (B and V).

To investigate whether high hepatic GCGR expression regulated physiological thermoregulation in *P. vlangalii*, we conducted liver in situ injections to deliver shRNA-AAV-*GCGR*, targeting endogenous GCGR (Fig. 2A). Compared with AAV-null controls, shRNA-AAV-*GCGR*–injected lizards showed remarkably decreased body temperature, blood glucose, and cyclic adenosine monophosphate (cAMP) levels at 4°C, whereas GCG levels did not differ between the two groups (Fig. 2, B to E, and fig. S3A). Downstream gene expression analysis of quantitative polymerase chain reaction (qPCR) revealed that hepatic *GCGR* knockdown reduced expression of thermogenesis (*PGC1A*, *FGF21*, and *PPARA*), glycogenolysis (*G6PC2* and *G6PC3*), as well as mitochondria-associated genes [cytochrome c1 (*CYC1*) and NADH dehydrogenase iron-sulfur protein 3 (*NDUFS3*)] (Fig. 2, G and H, and fig. S3B). Meanwhile, the genes related to lipid metabolism, i.e., lipid synthesis–related genes [fatty acid synthase (*FASN*) and acetyl–coenzyme A carboxylase (*ACACA*)], increased in the shRNA-AAV-*GCGR* group, and the genes related to lipid oxidation [carnitine palmitoyltransferase 1A (*CPT1A*) and carnitine palmitoyltransferase 1B (*CPT1B*)] decreased in the shRNA-AAV-*GCGR* group (Fig. 2H), indicating that pv*GCGR* can effectively regulate lipid metabolism levels of bodily functions at low temperatures. Also, hepatic *GCGR* knockdown in *P. vitticeps* and *P. erythrurus* showed the same phenotype (Fig. 2, I to V, and fig. S3, C to H).

**Fig. 3.**
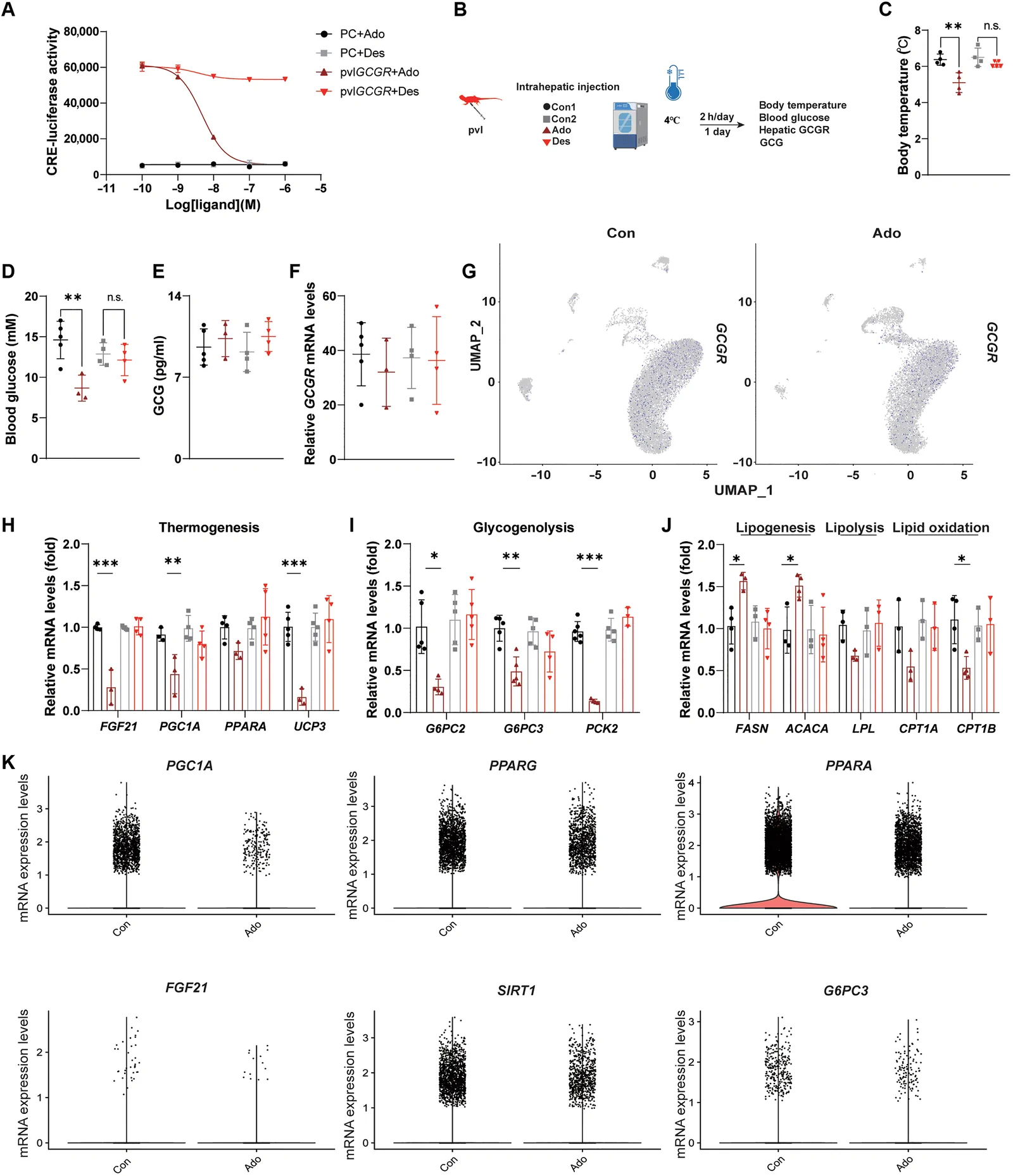
Inhibiting GCGR constitutive activity reduces lizard thermogenesis ability. (**A**) Effects of two allosteric GCGR inhibitors on the constitutive activity of *P. vlangalii* GCGR, Ado (dark red), and Des (red); PC corresponds to empty vector controls with Ado (black) or Des (gray). (**B**) After intrahepatic injection with Ado and Des, *P. vlangalii* was directly exposed to 4°C for 2 hours. (**C** to **F**) Body temperature was measured after the cold exposure, and after euthanizing, the following parameters were determined: body temperature (*n* = 4 to 5) (dark red symbols represent the Ado, and red symbols represent the Des groups; black and gray symbols represent the respective solvent control groups) (C), blood glucose (*n* = 3 to 5) (D), GCG content in serum (*n* = 3 to 5) (E), and hepatic *GCGR* mRNA expression (*n* = 4 to 5) (F). (**G**) UMAP diagram demonstrating the major distribution of *GCGR* mRNA in hepatocyte nuclei (*n* = 1). (**H**) Hepatic expression of thermogenesis-related genes (*PGC1A*, *UCP3*, *FGF21*, and *PPARA*) (*n* = 3 to 5), glycogenolysis-related genes (*G6PC2*, *G6PC3*, and *PCK2*) (*n* = 3 to 5) (**I**), and lipid metabolism–related genes (*FASN*, *ACACA*, *LPL*, *CPT1A*, and *CPT1B*) (*n* = 3 to 5) (**J**). (**K**) Differential thermogenesis-related gene expression was analyzed in the control and Ado hepatocyte groups of *P. vlangalii*. For abbreviations of gene symbols, refer to table S1. *P < 0.05; **P < 0.01; ***P < 0.001. Data are represented as means ± SD, analyzed using a one-way ANOVA followed by Tukey’s post hoc test (C to J).

To further confirm that constitutively active GCGR regulates physiological thermoregulation in *P. vlangalii* liver, we chose two different allosteric inhibitors: adomeglivant (Ado), which inhibits the constitutive activity of lizard GCGR through the G_s_ pathway (*31*) (Fig. 3A), and [Des-His^1^, Glu^9^]-glucagon amide (Des), which primarily inhibits GCGR via the G_q_ pathway (*31*) (Fig. 3A). Our structural modeling reveals distinct binding modalities dictated by the steric bulk and physicochemical properties of the respective ligands. The small molecule Ado penetrates deeply into the transmembrane (TM) bundle. In contrast, the bulky peptide ligand Des engages a more expansive surface area at the extracellular vestibule, forming extensive contacts with the extracellular loops and the top of the TM helices (fig. S4, A and B). Ado-treated *P. vlangalii* exhibited lower body temperature, blood glucose, and cAMP levels compared to the control group under cold conditions (Fig. 3, B to D, and fig. S3I), whereas GCG levels and GCGR expression remained unchanged (Fig. 3, E to G). Downstream analysis showed that the expression levels of PEPCK (*PCK2*, *G6PC2*, *G6PC3*, *PGC1A*, and *FGF21*), uncoupling protein 3 (*UCP3*), and *CYC1* mRNAs related to energy metabolism, gluconeogenesis, and mitochondrial oxidative phosphorylation remarkably decreased in the liver (Fig. 3, H and I, and fig. S3J). Meanwhile, the genes related to lipid metabolism, i.e., lipid synthesis–related genes (*FASN* and *ACACA*), increased in the Ado group, and the genes related to lipid oxidation (*CPT1B*) decreased in the Ado group (Fig. 3J). In contrast, *P. vlangalii* treated with Des did not exhibit a reduction in body temperature and cAMP levels, as well as expression of the aforementioned mRNAs (Fig. 3, B to J, and fig. S3, I and J).

Single-cell nuclear RNA sequencing (snRNA-seq) was performed on *P. vlangalii* liver samples from both control (*n* = 1) and Ado-treated (*n* = 1) groups. After integration using canonical correlation analysis (CCA), all high-quality cells were classified into six distinct cell types based on canonical markers (fig. S5, A to C). Among these, hepatocytes constituted the predominant cell population, accounting for over three-quarters of all cells (fig. S5B). The single-cell results demonstrated that GCGR was primarily expressed in hepatocytes (Fig. 3G). The energy metabolism–related genes (*PGC1A*, *PPARG*, *PPARA*, and *FGF21*, sirtuin 1, *SIRT1*, and *G6PC3*) had a certain downward trend in the Ado group and that the up-regulated genes in the Ado group were enriched in genes involved in lipid metabolism–related pathways (Fig. 3K and fig. S5D).

### Overexpression of constitutively active GCGR increases body temperature in lizards lacking thermoregulatory capacity

**Fig. 4.**
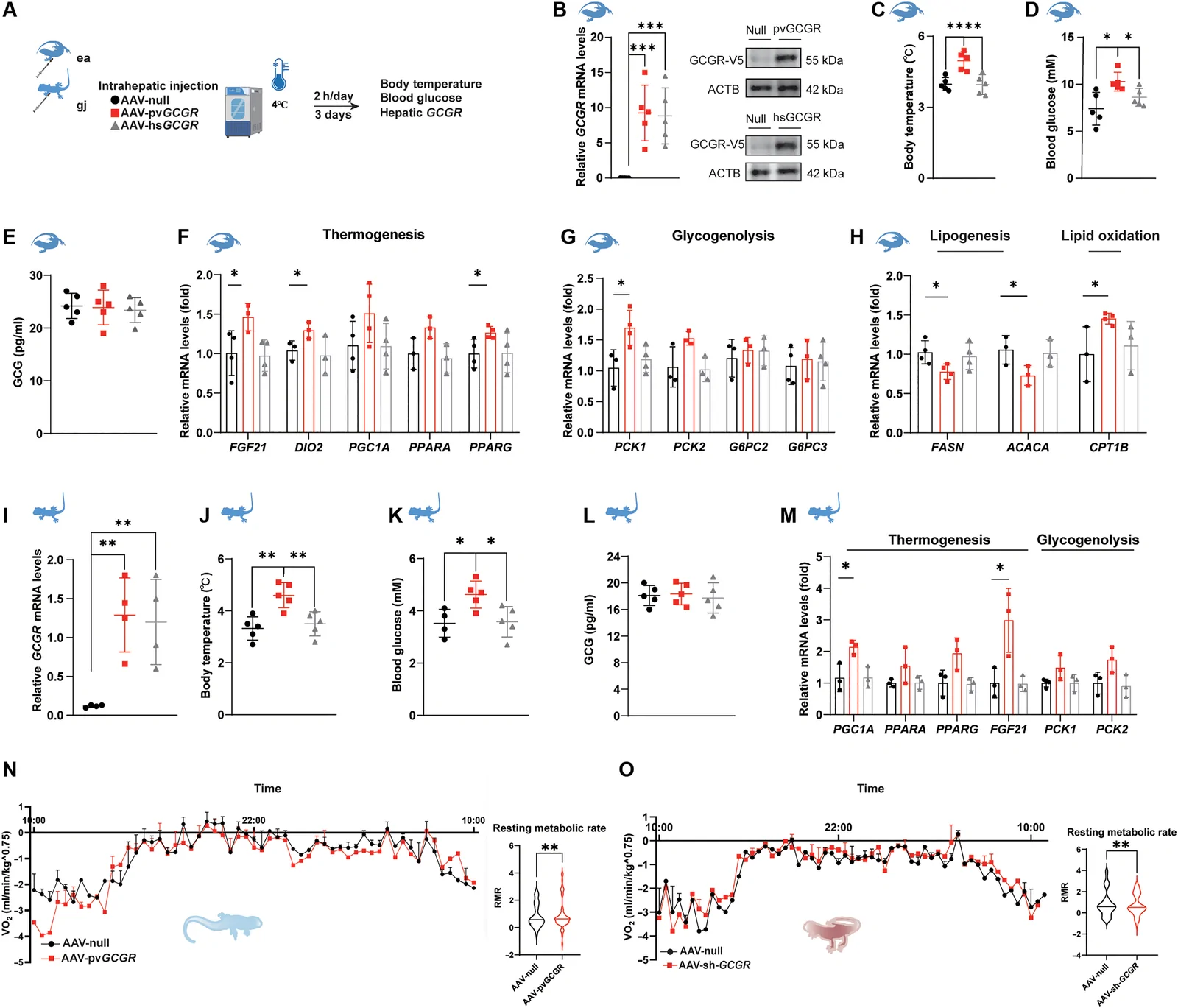
GCGR overexpression increases body temperature in thermoregulation-deficient lizards. (**A**) *E. argus* and *G. japonicus* received intrahepatic injections of AAV-null, AAV-hs*GCGR*, and AAV-pv*GCGR* (including the C-terminal V5 tag), followed by 2 hours of exposure to 4°C and 22 hours at 25°C. After the third cold-period, temperature was measured, followed by decapitation and measurements of the following parameters: (**B** to **E**) *E. argus* hepatic pv*GCGR* and hs*GCGR* exogenous mRNA expression (left; *n* = 5) and hepatic V5-tagged GCGR exogenous protein expression (right; *n* = 1) (B), body temperature (*n* = 5) (C), blood glucose levels (*n* = 4) (D), and GCG levels (E). (**F** to **H**) Hepatic expression of thermogenesis-related genes (*PGC1A*, *DIO2*, *FGF21*, *PPARA*, and *PPARG*) (*n* = 3 to 4) (F), glycogenolysis-related genes (*G6PC2*, *G6PC3*, *PCK1*, and *PCK2*) (*n* = 3 to 4) (G), and lipid metabolism–related genes (*FASN*, *ACACA*, and *CPT1B*) (*n* = 3 to 4) (H). (**I** to **L**) *G. japonicus* hepatic *GCGR* mRNA expression (*n* = 4) (I), body temperature (*n* = 4 to 5) (J), blood glucose levels (*n* = 4 to 5) (K), and GCG levels (L). (**M**) *G. japonicus* hepatic expression of thermogenesis-related genes (*PGC1A*, *FGF21*, *PPARA*, and *PPARG*) (*n* = 3) and glycogenolysis-related genes (*PCK1* and *PCK2*) (*n* = 3). Black represents the AAV-null group, red represents the AAV-pv*GCGR* group, and gray represents the AAV-hs*GCGR* group. (**N**) Oxygen consumption (VO_2_) in *E. argus* (left; *n* = 3) was measured 2 days after AAV-pv*GCGR* injection and resting metabolic rate (RMR) (right). (**O**) Oxygen consumption (VO_2_) in *P. vlangalii* (left; *n* = 3) 2 days after shRNA-*GCGR* and RMR (right). *E. argus* and *P. vlangalii* were housed at 20°C (lower than ambient temperature). *P < 0.05; **P < 0.01; ***P < 0.001. Data are represented as means ± SD, analyzed using a one-way ANOVA followed by Dunnett’s post hoc test (B to M) and unpaired Student’s *t* test (N and O).

To investigate the expression of the above identified regulatory genes involved in energy metabolism under low temperatures, we performed in situ injections of overexpression vectors AAV-pv*GCGR*, AAV-hs*GCGR*, and AAV-null into the liver of *E. argus*, a species with low GCGR expression and without physiological thermoregulation at low temperatures (Fig. 4A). As seen in Fig. 4B, GCGR mRNA expression was higher in the pv*GCGR* group at 4°C. The body temperature, blood glucose, and cAMP levels in the pv*GCG*R group were notably higher compared to those from the AAV-null group and the hs*GCGR* group, but the GCG levels remained the same (Fig. 4, C to E, and fig. S3K). Downstream expression of genes related to thermogenesis [*FGF21*, iodothyronine deiodinase 2 (*DIO2*), and *PPARG*], blood glucose metabolism [*PEPCK 1* (*PCK1*)], cold sensation (transient receptor potential cation channel subfamily M member 8, alternatively termed menthol receptor 1, *TRPM8*, or *CMR1*), and mitochondrial energy metabolism [*NDUFS3*, *CYC1*, and cytochrome c oxidase subunit 4 isoform 1 (*COX4I1*)] were also elevated (Fig. 4, F and G, and fig. S3, L and M). The genes related to lipid metabolism, i.e., lipid synthesis–related genes (*FASN* and *ACACA*), decreased in the AAV-pv*GCGR* group, and the genes related to lipid oxidation (*CPT1B*) increased in the AAV-pv*GCGR* group (Fig. 4H). Last, we overexpressed pv*GCGR* in *Gekko japonicus* liver and observed similar phenotype and gene expression changes compared to those from the AAV-null and hs*GCGR* group (Fig. 4, I to M, and fig. S3, N to P). Body temperature, blood glucose, and cAMP levels in the pv*GCGR* group were higher compared to those from the AAV-null group and hs*GCGR* group, but the GCG levels remained the same at 4°C (Fig. 4, J to L, and fig. S3N). In the AAV-pv*GCGR* group, the mRNAs transcribed from the downstream genes related to glycogenolysis, thermogenesis, cold sensation, and mitochondrial energy metabolism were also higher than those from the AAV-null group and hs*GCGR* group (Fig. 4M and fig. S3, O and P).

### Hepatic overexpression of *P. vitticeps* GCGR increases body temperature and energy metabolism in mammals at low temperatures

**Fig. 5.**
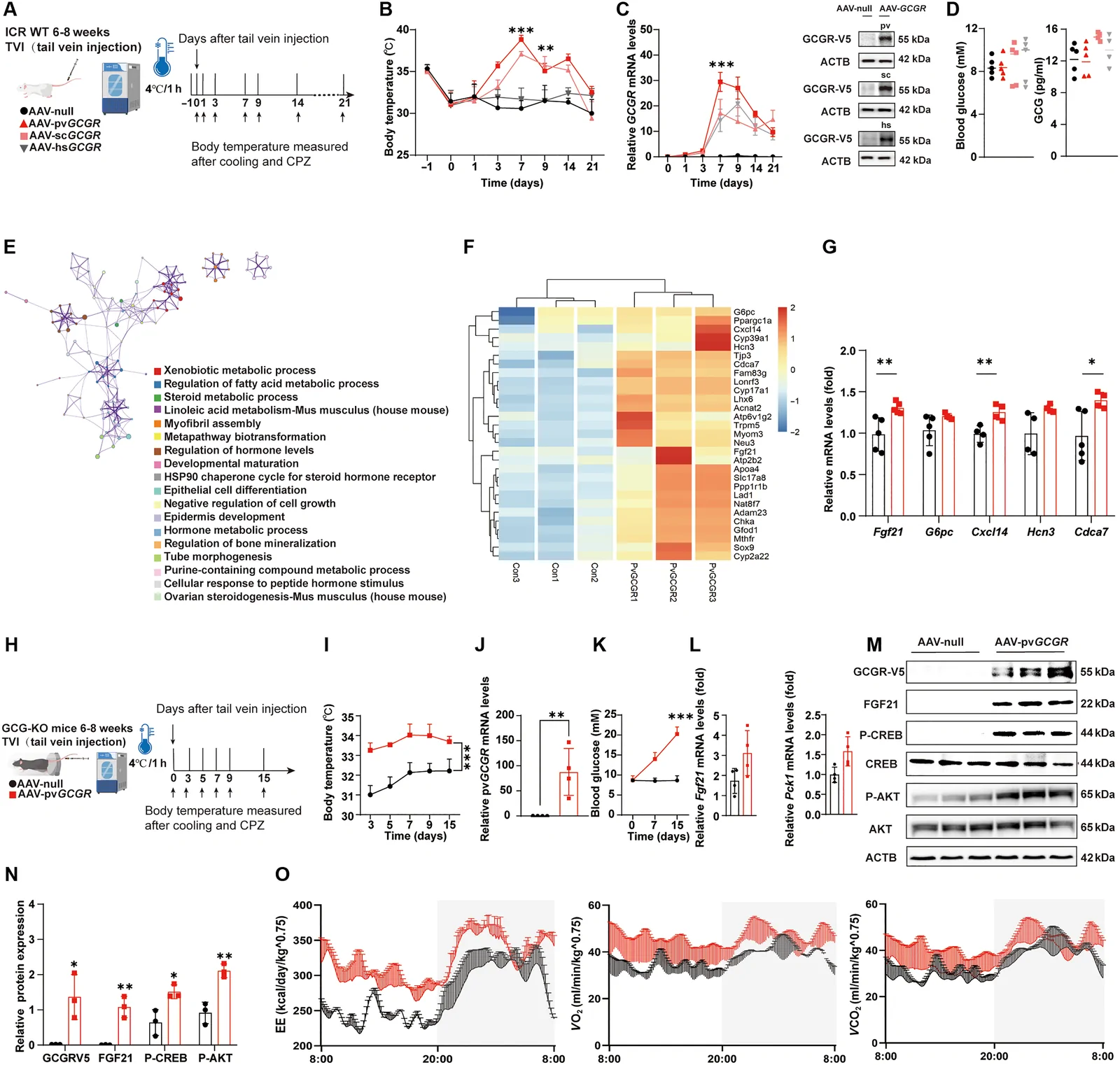
Lizard GCGR overexpression elevates body temperature and energy metabolism in mice. (**A**) Tail vein injection of AAV-null, AAV-hs*GCGR* (1.5 × 10^11^ v.g.), AAV-pv*GCGR* (1.25 × 10^11^ v.g.), or AAV-sc*GCGR* (5 × 10^10^ v.g.) constructs into male ICR mice (*n* = 21 per group). On day −1, mice received 1 hour 4°C exposure followed by temperature assessment. On days 0, 1, 3, 7, 9, 14, and 21 postinjection, CPZ (2.5 mg/kg, intraperitoneally) was administered before 1 hour 4°C exposure, after which temperature was measured. WT, wild type. (**B** to **D**) Body temperature (*n* = 3 to 5) (B) and hepatic *GCGR* mRNA expression in mice (left; *n* = 3 to 5) and hepatic exogenous V5-tagged GCGR protein expression (right; *n* = 1) (C), blood glucose (left; *n* = 5), and GCG levels (right; *n* = 3 to 5) at 7 days postinjection (D). (**E** to **G**) Liver RNA-seq was performed on AAV-null (con; *n* = 3) and AAV-pv*GCGR* (*n* = 3) mice 7 days postinjection after 1 hour 4°C exposure. The enriched pathways (E) and heatmap (F) of DEGs and hepatic *Fgf21*, *G6PC*, *Cxcl14*, *Hcn3*, and *Cdca7* mRNA expression levels in mice (*n* = 4 to 5) (G). (**H** to **O**) Male *GCG*-KO mice were injected (tail vein) with AAV-null (*n* = 4) and V5-tagged AAV-pv*GCGR* (*n* = 4) (1.25 × 10^11^ v.g.) (H). Mice received CPZ (2.5 mg/kg, intraperitoneally) on days 3, 5, 7, 9, and 15, followed by 1 hour 4°C exposure and body temperature measurement (*n* = 3 to 4) (I), and after euthanizing, hepatic exogenous pv*GCGR* mRNA expression (*n* = 4) (J), blood glucose (*n* = 4) (K), *Fgf21* (left; *n* = 4), and *Pck1* (right; *n* = 4) mRNA levels (L) were measured. Exogenous V5-GCGR, endogenous FGF21, and total/phosphorylated AKT and CREB were assessed by Western blotting (*n* = 3) (M) and Western blot quantification (*n* = 3) (N). Energy consumption (*EE*), oxygen consumption (*V*O_2_), and carbon dioxide production (*V*CO_2_) in *GCG*-KO mice were measured 7 days after AAV-pv*GCGR* injection at the room temperature (*n* = 3) (O). Data are means ± SD, analyzed using a two-way ANOVA (B, C, I, and K), one-way ANOVA followed Tukey’s post hoc test (D), and unpaired Student’s *t* test (G, J, L, N, and O).

To assess whether *P. vitticeps* GCGR can enhance body temperature and energy metabolism at low temperatures in mammals, we used AAV vectors to overexpress pv*GCGR* and *Serinus canarius GCGR* (sc*GCGR*) in 8-week-old male Institute of Cancer Research (ICR) mice that were pretreated with chlorpromazine (CPZ) to reduce the ability of hypothalamic thermoregulation (*35*) (Fig. 5A). Mice injected with CPZ (2.5 mg/kg) dropped their temperatures to ∼30°C, with no significant increase in blood glucose levels (fig. S6C). Therefore, we chose 2.5 mg/kg for subsequent experiments. Before exposing the mice to 4°C, we intraperitoneally injected them with CPZ (2.5 mg/kg) and measured their body temperature 1 hour after placing them in a 4°C environment. As expected, pv*GCGR* was highly expressed in the liver and body temperatures (35°C) were notably elevated, compared to AAV-null and hs*GCGR* groups (30°C), without elevating blood glucose or GCG levels, as observed in ICR negative control mice (Fig. 5, B to D). This effect persisted for ∼21 days (Fig. 5C). Notably, avian (canary) sc*GCGR* overexpression showed a similar effect to pv*GCGR* (Fig. 5, B to C). RNA-seq data further indicated that the differentially expressed genes (DEGs) in the pv*GCGR* group were mainly involved in lipid metabolism and HSP90 pathways, as well as up-regulated *Fgf21*, a gene that acts downstream of *GCGR* and is associated with thermogenesis (Fig. 5, E and F). We validated some DEGs from bulk RNA-seq using qPCR, and the results were consistent with the sequencing data. Specifically, the thermogenesis-associated gene *Fgf21* showed up-regulated expression (Fig. 5G).

### *P. vitticeps* GCGR body temperature regulation is ligand independent

On the basis of our observation that lizard GCGR overexpression in mice did not essentially alter GCG concentrations (Fig. 5D), we suggest that the GCGR-induced body temperature increase in lizards at low temperatures is GCG independent. Therefore, we verified this in *GCG*-KO (knockout) mice to ascertain the functionality of pv*GCGR* in the liver. These mice were kept on a normal diet and received tail vein injections of AAV-pv*GCGR*. Before exposure to 4°C for 1 hour, the *GCG*-KO mice were intraperitoneally injected with CPZ (2.5 mg/kg) to eliminate neuromodulation effects of body temperature (*36*), after which their rectal temperature was measured (Fig. 5H). The results exhibited higher blood glucose levels and increased body temperature at 4°C (Fig. 5, I to K). Moreover, mRNA expression of the downstream thermogenesis-related gene *FGF21* and glucose metabolism–related gene *PCK1* was also increased in the pv*GCGR* group (Fig. 5L) and confirmed by Western blotting of the GCGR protein (Fig. 5, M and N). Furthermore, overexpression of pv*GCGR* led to increased phosphorylation of CREB and AKT in the liver (Fig. 5, M and N).

We also performed a ligand stimulation assay in ICR mice using IUB288, an agonist of GCGR, which can further enhance the G_s_ pathway in HEK293T cells overexpressing pv*GCGR* or pvl*GCGR* (fig. S6A). After intraperitoneal injection of IUB288, mice were exposed to 4°C for various times. Compared to the saline-injected control group, IUB288 substantially elevated body temperature following 2 hours of cold exposure (fig. S6B). Furthermore, we found that the agonist IUB288 considerably increased the body temperature and blood glucose of *E. argus* after 1 hour exposure to 4°C (fig. S6, D and E). Last, the expression of genes related to thermogenesis [*FGF21*, *DIO2*, and uncoupling protein 2 (*UCP2*)], glycogenolysis (*G6PC2* and *PCK1*), lipolysis [lipoprotein lipase (*LPL*) and adipose triglyceride lipase (*ATGL*)], and lipid oxidation (*CPT1B*) increased in the livers of IUB288-treated lizards (fig. S6, F to I), suggesting that the lizard GCGR exerts a thermogenic effect through its expression and constitutive activity, which can also be activated by the ligand.

### Constitutively active GCGR increases basal metabolic rate

Following the close correlation between energy metabolism and body temperature (*37*), we further investigated the relationship between constitutively active GCGR in the liver and the basal metabolic rate (BMR) in *GCG*-KO mice. As shown in Fig. 5B, mice can reach the highest GCGR expression and the highest temperature on day 7 post–pv*GCGR* injection (Fig. 5B). Therefore, we chose mice on day 7 after AAV injection for intraperitoneal CPZ administration, followed by placement into metabolic cages. Indirect calorimetry analysis revealed that the pv*GCGR*-overexpressing mice revealed higher oxygen consumption (*V*O_2_) and carbon dioxide production (*V*CO_2_) compared to the control group, with increased energy expenditure rate during both light and dark phases (Fig. 5O). Similar results could also be seen in the pv*GCGR*-overexpressing *E. argus* lizard (Fig. 4N). In contrast, *GCGR* knockdown in *P. vlangalii* lizards reduced VO_2_ levels (Fig. 4O).

## DISCUSSION

Endotherms produce heat internally through metabolic processes and regulate their temperature through various mechanisms, such as BMR, shivering thermogenesis, nonshivering thermogenesis (*38*, *39*), and vasodilation and vasoconstriction mechanisms (*40*). In contrast, ectotherms (e.g., reptiles, amphibians, and most fish) primarily rely on external environmental heat sources and behavioral thermoregulation to regulate their body temperature (*41*). However, there is also a large body of research showing that reptiles such as lizards can adapt to low temperatures through different physiological mechanism (*42*). Research indicates that certain, but not all, lizards have an enhanced ability to regulate their body temperature in response to environmental temperature fluctuations (*43*). This thermoregulatory capacity allows their body temperature to be slightly higher than the ambient temperature under low-temperature conditions (*44*). For example, studies have shown that the lizard *Zootoca vivipara*, formerly *Lacerta vivipara*, exhibits a 20% increase in anaerobic metabolism during winter compared to other seasons. This is mainly due to the activation of the lactic acid fermentation pathway leading to an increase in lactate concentration (>34% in winter). In addition, glucose levels increase by about 245% in winter, serving as an antifreeze and metabolic substrate (*45*). Aerobic metabolism gradually decreases in late fall, resulting in resting VO_2_ at 17°C that stabilizes at ∼20% of resting values throughout winter dormancy. In the annual cycle of the tegu, *Salvator merianae*, this period of metabolic suppression is considered a form of active winter sleep (*46*). Our previous work established that constitutively active GCGR modulates blood glucose and metabolic rates in birds and the metabolic rate of the family Iguanidae is higher than that of other reptilian families (*31*). In the present work, we found that most Iguanidae species can slightly elevate their body temperatures in cold environments. This physiological thermoregulation is primarily mediated by the hepatic constitutively active GCGR. This indicates that lizards might regulate thermoregulation through up-regulation of constitutively active GCGR expression in the liver. Overall, cold exposure increases hepatic GCGR expression in lizards, which constitutively activates the G_s_ signaling pathway, leading to elevated cAMP levels. Elevated cAMP concomitantly promotes glycogenolysis to increase blood glucose levels while up-regulating thermogenic gene expression to elevate body temperature. Our study offers yet unknown insights into expression regulation of constitutively active GPCR into physiological adaptations responding to environmental changes in ectothermic animals.

GPCRs have emerged as pivotal drug targets for multiple chronic diseases, including obesity and diabetes (*47*). GCGR, a class B GPCR, predominantly expressed in the liver and kidney, is primarily activated by glucagon. Its activity is well established in regulating the pathogenesis of diabetes and obesity-associated glucose dysregulation (*48*). Glucagon is a crucial regulator of glucose and lipid homeostasis, as well as whole-body energy balance, by suppressing food intake and stimulating energy expenditure and promoting hepatic glucose production (*49*). In recent years, numerous dual agonists of GCGR and GLP1R have been developed for obesity treatment, demonstrating simultaneous improvement in glycemic control (*50*). For example, the dual GLP-1/GCGR agonist G49 reduces lipid by enhancing energy metabolism mediated by FGF21 (*51*). Although previous research has primarily focused on the effects of agonists on GCGR, the intrinsic role of the receptor itself in lipid metabolism and glucose homeostasis remained unclear.

Numerous GPCRs are in equilibrium between active or inactive (*52*). For example, metabotropic glutamate receptors (mGlus) coupling to effector molecules (including G proteins or β-arrestins) not only mediate downstream effectors but also determine the activity of mGlus itself. These effectors coupled to mGlus and the activation of downstream pathways can be regulated by mutual interactions between binding molecules, including kinases, phosphatases, and proteins (*53*). One of our published works demonstrates that the avian GCGR exhibits high constitutive activity, which sustains hyperglycemia to meet the energy demands of flight (*54*). Notably, it also reduces adipose mass in both avian and murine models by enhancing energy expenditure through the up-regulation of *Pgc1a*, *Pparg*, and *G6pc*. Similarly, the GCGR in lizards displays elevated constitutive activity; however, its physiological effects in reptilian species remained unexplored. Here, we found that overexpression of constitutively active pv*GCGR* in the liver of nonthermoregulating lizards up-regulated several pathways, including glucose regulation (*G6PC* and *PCK*), thermogenesis (*FGF21*), and lipid metabolism, as well as *TRPM8* involved in cold sensation and genes associated with mitochondrial energy metabolism (*CYC1*, *NDUFS3*, and *COX4I1*). Consistent with previous reports (*31*, *51*), this extensive metabolic reprogramming ultimately led to higher standard metabolic rates (SMRs) in ectotherms and elevated BMRs in endotherms, thereby increasing overall energy metabolism and body temperature. Conversely, suppressing GCGR expression in the liver of thermoregulating lizards yielded the opposite effect. These findings reaffirmed the significant regulatory role of GCGR in nonshivering thermogenesis in the lizard liver and indicated that high expression levels of constitutively active GCGR can regulate body temperature, blood glucose, and energy metabolism. Furthermore, we found that the role of lizard GCGR in thermoregulation could only be inhibited by Ado, a specific antagonist of GCGR constitutive activity, indicating that constitutively active GCGR is a critical thermogenic gene in reptiles. These results indicate that constitutively active GCGR in the liver can mediate physiological thermoregulation by modulating the expression of hepatic energy metabolism–related genes in lizards. Together, these findings demonstrate that hepatic expression of constitutively active GCGR in mice consistently increased body temperature at low temperatures.

Hepatic expression of mammalian *GCGR*, which lacks constitutive activity, did not elevate body temperature in mice. In contrast, overexpression of the lizard pv*GCGR* markedly increased metabolic rate and body temperature under cold conditions when compared to the AAV-null group, further corroborating the thermogenic role of constitutively active GCGRs. These data indicated that constitutively active GCGR plays a pivotal role in regulating heat production in ectothermic animals, contributing to their ability to maintain body temperature and overexpression of constitutively active GCGR expressed in the liver can enhance the BMR of mice. This constant activation may be particularly crucial for these ectothermic animals in adapting to varying environmental conditions, allowing for optimized energy utilization and homeostasis maintenance in diverse ecological habitats.

Although we found that GCGR regulates thermogenesis, circulating GCG levels remained unchanged during cold stimulation and even after pv*GCGR* overexpression, suggesting that hepatic GCGR expression in lizards may be involved in thermoregulation at low temperatures, primarily through higher GCGR expression level and constitutive activity rather than the GCG ligand. Specifically, low temperatures up-regulated lizard GCGR expression, promoting thermogenesis and glycogenolysis alongside enhanced lipid oxidation, without altering GCG levels (Fig. 1, J to P). In both species, IUB288 elevated body temperature and blood glucose levels by modulating thermogenic gene expression, although the underlying mechanisms likely differ. The thermoregulatory role of IUB288 in mice aligns with a prior report (*25*). In *GCG*-KO mice, overexpression of pv*GCGR* also induced BMR changes and increased body temperature and *Fgf21* levels under cold conditions, whereas other elevated genes without previously reported thermogenic functions require further functional validation. This suggests that, although a GCGR agonist could elevate body temperature and blood glucose in lizards, GCGR-mediated thermoregulation in lizards occurs via a ligand-independent pathway.

In lizards, thermoregulatory capacity is associated not only with critical temperatures but also with the thermal reaction constant, namely, the warming/cooling rate (*55*, *56*). Generally, the larger the thermal reaction constant, the longer it takes for an organism to warm up or cool down, and the more difficult it is for ectotherms to alter their body temperature under the same experimental conditions, and vice versa. Accordingly, we examined the thermal reaction constants of different lizard species under experimental conditions. Our results showed that, despite its larger body mass, *P. vitticeps* exhibited the smallest thermal time constant, indicating a higher thermoregulatory performance. Body mass is a key physiological factor influencing the warming and cooling time constants, and previous studies have suggested that the physiological thermoregulation ability of lizards is inversely proportional to body size (*57*, *58*). We speculate that the relatively small body size of *Eremias przewalskii* and *Scincella modesta* had a strong impact on their thermal reaction constants, resulting in values second only to *P. vitticeps*. Although *P. vlangalii* showed reduced thermal responsiveness due to its larger body size, it was still able to maintain a body temperature 5.5°C above the 4°C ambient temperature, unlike *S. modesta*, which could not exceed 4°C. Multivariate analysis therefore confirmed that *P. vlangalii* retains a relatively strong thermoregulatory capacity. In this study, we demonstrated that constitutively active GCGR contributes to thermoregulation in lizards under cold conditions; however, whether it also regulates their warming/cooling rates remains to be investigated.

**Fig. 6.**
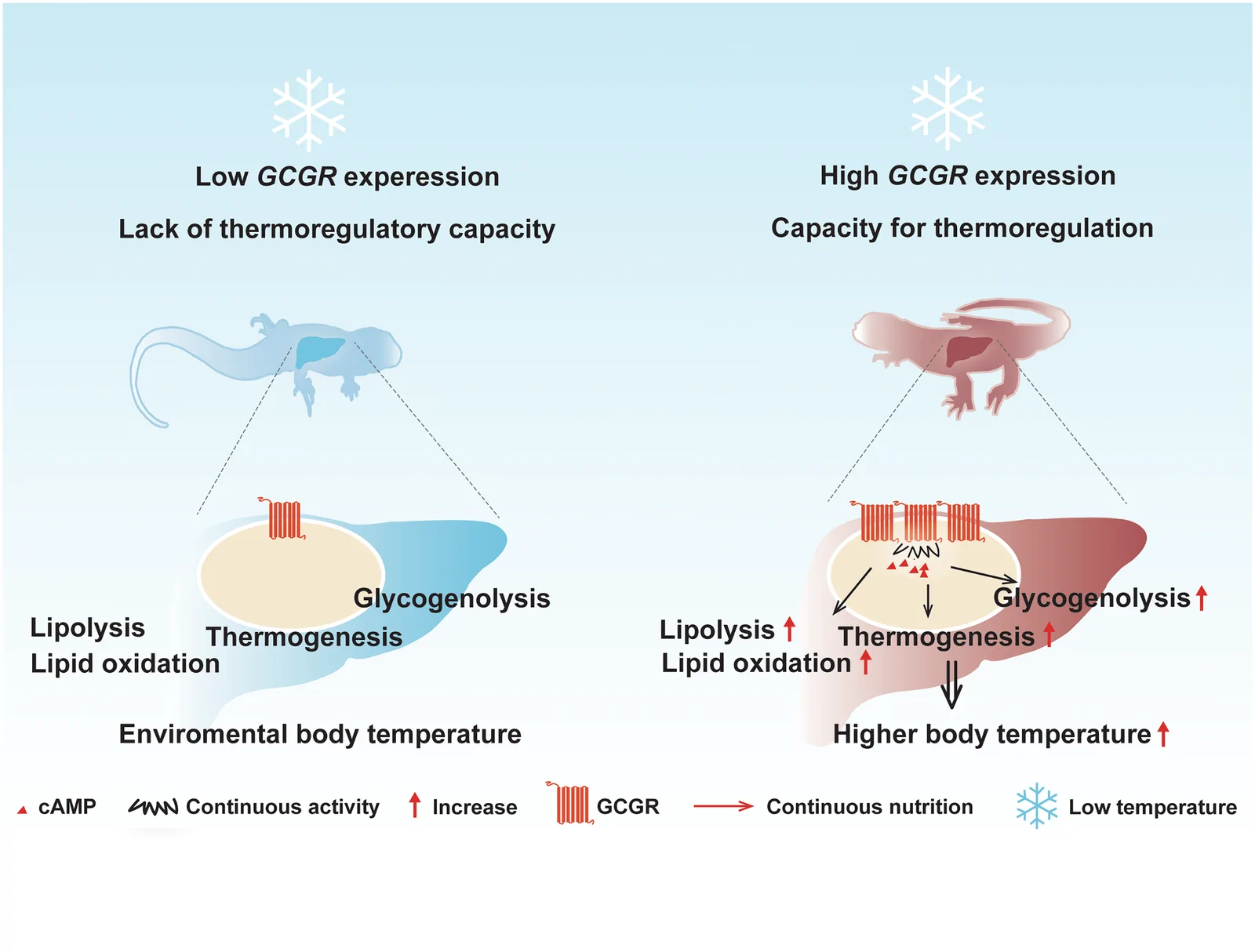
GCGR regulates the thermogenesis effects of lizards. GCGR exhibits ligand-independent constitutive activity of the G_s_ signaling pathway. Under cold exposure (4°C), hepatic GCGR expression in lizards positively correlates with blood glucose levels and thermoregulatory capacity. In Iguanidae, such as *P. vlangalii* (right), elevated hepatic GCGR expression under cold stress constitutively activates the G_s_ signaling pathway, enhancing energy, glucose, and lipid metabolism, thereby promoting thermogenesis and elevating body temperature. Conversely, lizards such as *E. argus* (left) display lower hepatic GCGR expression, leading to reduced metabolic rates and an inability to increase body temperature through thermogenesis under identical conditions. Red upward arrows denote up-regulation of downstream metabolic genes, and red triangles represent cAMP, the key second messenger in the GCGR-G_s_ signaling cascade.

On the basis of our experimental findings, we propose a mechanistic model illustrating the molecular basis of thermoregulatory capacity in lizards under cold stress (Fig. 6). Central to this model is the critical role of hepatic GCGR expression and its downstream signaling. In species capable of thermoregulation, cold environments trigger high GCGR expression in the liver. This elevated receptor level, coupled with its continuous constitutive activity, results in accumulation of intracellular cAMP. The amplified cAMP signaling cascade subsequently up-regulates key energy-yielding metabolic pathways, specifically lipolysis, lipid oxidation, and glycogenolysis. The synergistic enhancement of these pathways provides the necessary substrates to fuel robust thermogenesis, ultimately allowing the organism to maintain a higher body temperature despite cold surroundings. Conversely, in species lacking thermoregulatory capacity, low GCGR expression fails to efficiently activate this metabolic network, resulting in a passive body temperature that reflects the cold environment. Collectively, this model (Fig. 6) provides a comprehensive overview of how hepatic GCGR-mediated metabolic reprogramming serves as a fundamental strategy for thermal adaptation.

In conclusion, constitutively active GCGR drives the physiological thermoregulation in lizards, offering insights into how the expression and regulation of constitutively active GPCRs facilitate physiological adaptations to environmental changes.

## MATERIALS AND METHODS

### Reagents

GCGR overexpression and AAV serotype 2/8 for knockdown experiments was purchased from Heyuan Biotechnology Co. Ltd. (Shanghai, China). The GCGR agonist IUB288 (HD29) was purchased from Jill Biochemical Co. Ltd. (Shanghai, China). The GCGR inhibitor Ado (LY2409021) was purchased from Shanghai Taoshu Biotechnology Co. Ltd., and Des and CPZ were purchased from MedChemExpress Biotechnology Co. Ltd. (USA).

### Animals

The *P. vlangalii* and *P. erythrurus* specimen were caught in Bayannur City, Inner Mongolia, in July. *P. vitticeps* were purchased from Hui’an County, Fujian Province. *E. argus* and *G. japonicus* were purchased from Julu County, Xingtai City, Hebei Province and Luzhai County, Guangxi Zhuang Autonomous Region, respectively. All lizards for experiments are male and were housed in laboratory enclosures under controlled conditions: a 12-hour light/12-hour dark photoperiod provided by temperature-regulated lamps usually at 25°C. *P. vitticeps*, *E. argus*, *P. vlangalii*, and *P. erythrurus* were housed in enclosures with fine sand substrate. *G. japonicus* was housed on a substrate of coconut soil and moss. All species were allowed to acclimate to these laboratory conditions for at least 3 days before experimentation. Lizards were fed mealworms (*Tenebrio molitor*) and water regularly every day, with vitamin D powder added to the water to prevent calcium deficiency.

Male ICR strain mice aged 6 to 8 weeks were purchased from Wukong Biotechnology Co. Ltd. (Jiangsu, China). Heterozygotic *GCG*-KO mice were purchased from GemPharmatech Co. Ltd. (China). Mice were housed under specific pathogen–free (SPF) conditions at 25°C and provided with standard chow (XT93M, Jiangsu Synergy Pharmaceutical Bio-engineering Co. Ltd., Jiangsu, China), and the experimental treatments were begun after the mice adapted to the new environment for 1 week. All animal experimental and care procedures were performed in accordance with the guidelines of Animal Management Regulations of the Ministry of Health, China (Document No. 55, 2001) and were approved by the Nanjing Normal University Animal Faculty (IACUC-20210233).

### AAV-mediated overexpression or shRNA-AAV–mediated knockdown and injection of substances

*P. vlangalii*, *P. vitticeps*, and *P. erythrurus* were divided into two groups: one was the virus control group and the other was the shRNA-AAV-*GCGR* [2.5 × 10^11^ vector genomes (v.g.)] group. *E. argus* and *G. japonicus* were divided into three groups: one was the empty virus control group, one was the group overexpressing *GCGR* with AAV-pv*GCGR* virus (1.5 × 10^11^ v.g.), and the third was the group overexpressing *GCGR* with AAV-hs*GCGR* virus (1.5 × 10^11^ v.g.). After 3 days of orthotopic liver injection, the lizards were placed in a 4°C incubator for 2 hours and the temperature was measured before decapitation to collect blood and relevant tissue. *P. vlangalii* was divided into four groups: both the control group [one was injected with 15% dimethyl sulfoxide (DMSO) and 15% Tween 80 dissolved in normal saline and the other was injected with 5% DMSO dissolved in normal saline], the treatment groups comprised the Ado (5 mg/kg) group, and the Des (4 μg/kg) group. *E. argus* was divided into two groups: one was the control group (injected with 5% DMSO dissolved in normal saline) and the other was the IUB288 (10 nmol/kg) group. The lizards were placed in a 4°C incubator for 2 hours after orthotopic liver injection. The cloacal temperature was measured before decapitation followed by collecting blood and relevant tissue.

ICR mice were divided into four groups for tail vein injection of AAV to express in the liver, namely, AAV-null, AAV-hs*GCGR*, AAV-sc*GCGR*, and AAV-pv*GCGR*. Mice were injected with CPZ intraperitoneally before being placed in a 4°C incubator for 1 hour on days −1, 0, 1, 3, 7, 9, 14, and 21 after tail vein injection, and their body temperatures were measured after cold exposure. Subsequently, blood was collected from the orbital sinus and the mice were decapitated to collect liver tissue.

*GCG-KO* mice were divided into two groups for tail vein injection of AAV to express in the liver, namely, AAV-null and AAV-pv*GCGR*. Mice were injected with CPZ intraperitoneally before being placed in a 4°C incubator for 1 hour on days −3, 5, 7, 9, and 15 after tail vein injection, and their body temperatures were measured after cold exposure. Subsequently, blood was collected from the orbital sinus and the mice were decapitated to collect liver tissue.

### GCG detection

Mouse blood samples were centrifuged at 5000 rpm for 20 min at 4°C to isolate serum. Serum GCG concentrations were measured using a mouse GCG enzyme-linked immunosorbent assay (ELISA) kit (Aifang Biological, China). For lizard samples, blood was centrifuged in the same way to obtain serum, and the GCG concentration was measured using a chicken GCG ELISA kit (Aifang Biological, China).

### RNA extraction, reverse transcription PCR, and qPCR

Total RNA was extracted from the liver tissues using TRIzol reagent (catalog no. CW0580S, Invitrogen, Jiangsu, China) according to the manufacturer’s instructions. RNA was reverse transcribed using a HiScript III RT SuperMix for qPCR (+gDNA wiper) (R223-01, Vazyme, Jiangsu, China) according to the manufacturer’s protocol, and qPCR was performed on a Bio-Rad CFX96 machine (QS5, Applied Biosystems, USA) with the 2×SuperFast Universal SYBR Master Mix (catalog no. CW3888M, CWBIO, Jiangsu, China). The mRNA levels of selected genes were calculated after normalization to β*-actin* by using the 2^−∆∆CT^ method. Primer sequences are listed in table S4.

### Immunoblotting/Western blotting analysis

Liver protein of lizards and mice was extracted using radioimmunoprecipitation assay lysis buffer (PC101, Shanghai Epizyme Biomedical Technology, Shanghai, China) containing protease inhibitors (GRF101, Shanghai Epizyme Biomedical Technology, Shanghai, China). Equal amounts of protein lysates were separated by SDS–polyacrylamide gel electrophoresis and transferred onto polyvinylidene difluoride membranes (Millipore, Billerica, MA, USA). Antibodies for detection are listed in table S5.

### Cell lines and cell transfection

AML12 and HEK293T cells were obtained from the National Collection of Authenticated Cell Cultures (Chinese Academy of Sciences, Shanghai, China). AML12 cells were cultured in medium (Gibco) containing Dulbecco’s modified Eagle’s medium (DMEM)/F-12, 10% fetal bovine serum (FBS), 0.5% insulin-transferrin-selenium (ITS-G) (100×), dexamethasone (40 ng/ml), and 1% penicillin/streptomycin (P/S). HEK293T cells were cultured in DMEM (Gibco) supplemented with 10% FBS, penicillin (100 IU/ml), and streptomycin (100 μg/ml). AML12 and HEK293T cells were cultured in an incubator containing 95% air and 5% CO_2_ at 37°C.

*GCGR* genes were synthesized by General Bio Co. Ltd. (Anhui, China) and Tsingke Biotechnology Co. Ltd. (Beijing, China) and then cloned into pcDNA3.1 with C- or N-terminal V5-His tags. When HEK293T and AML12 cells reached 80% confluency, the plasmids and empty vector plasmids were transfected with a concentration of ∼100 ng/μl for every plasmid. After 48 hours, the samples were collected, and RNA was extracted for measuring the expression of downstream genes.

### Luciferase assay and quantification of GCGR expression in cells by ELISA

To assess whether GCGR has constitutive activity, HEK293T cells were placed into a 24-well plate and at 60% confluency, and the *GCGR* plasmids and pcDNA3.1 were transfected with serum-free medium. Six hours posttransfection, the medium was replaced with a medium containing serum and the cells were cultured for another 24 hours before harvesting. Luciferase activities were determined using luciferase assay kits (Yeasen, Shanghai, China).

Twenty-four or 48 hours after transfection, the medium was removed and the cells were washed with phosphate-buffered saline (PBS). Cells were fixed with 4% paraformaldehyde (PFA) for 15 min and then washed three times with PBS. The cells were osmotically stabilized with 0.1% Triton X-100 for 15 min and then washed three times with PBS. The cells were blocked with 1% FBS (blocking buffer) or 1% bovine serum albumin (Beijing Solarbio Science & Technology Co. Ltd., China) for 1 hour. Cells were then incubated with primary antibody (anti-V5, 1:1000; Cell Signaling Technology, catalog no. 13202S, USA) diluted in blocking buffer for 2 hours. After three washes with blocking buffer, cells were incubated with secondary antibody [anti-mouse/rabbit horseradish peroxidase (HRP), 1:1000; Beyotime] diluted in blocking buffer for 30 min at room temperature. HRP activity was detected using a chemiluminescent substrate (SuperSignal, Vazyme, China) or 3,3ʹ,5,5ʹ-Tetramethylbenzidine (TMB) substrate solution (Beyotime, Shanghai, China). Luminescence signals were measured using a Synergy H1 Multi-Mode Microplate Reader (BioTek, USA).

### cAMP assay in the liver of lizards

Liver tissue cAMP levels in lizards were measured using commercial ELISA kits (Shanghai Enzyme-linked Biotechnology, China), all performed according to the manufacturer’s instructions.

### BRET assay

For the G_s_ pathway assay, HEK293 cells were seeded in a 6-well plate and incubated overnight. Then, cells were treated with PEI transfection reagent (YEASEN, 40816ES02) with 1.0 μg each of the receptor (GCGR), Gα-RLuc8, Gβ, and Gγ-GFP2. After 24 hours of transfection, cells were seeded into 96-well plates with white flat bottom. After 24 hours, the medium was removed. The plates were washed and replaced with Hanks’ balanced salt solution (HBSS) buffer. One hundred microliters of 5 μM coelenterazine 400a (Yeasen, Shanghai, China) was added, and plates were read immediately using emission filters set at 410 nm (Rluc8) and 515 nm (GFP2).

### Thermal reaction determination constant

For better control of ambient temperature, the elevated-temperature experiment was conducted in a thermostat-controlled chamber. Core body temperature (*T*_b_) was continuously recorded using a temperature probe inserted 1 cm into the cloaca (lizards) or rectum (mice). During the cooling and warming processes, *T*_b_ was recorded at every 5°C interval between 10° and 35°C. Thermal time constants for cooling and heating were calculated by performing linear regression of time (*t*, min) against lg|*T*_a_ – *T*_b_| (*T*_a_ is the ambient temperature, and *T*_b_ is the body temperature) and obtaining the slope of the linear equation.

### Bulk RNA-seq analysis

Total RNA was extracted from livers of ICR mice, quantified, and used for library construction and sequencing by Novogene Co. Ltd. (Beijing, China). The quality of the raw data was assessed using FastQC (http://www.bioinformatics.babraham.ac.uk/projects/fastqc/), and adapter sequences were removed with Trimmomatic (v.0.36). Clean reads were aligned to the *Mus musculus* genomes using HISAT2 (v.2.2.1) with default parameters. Paired-end reads were sorted into BAM format using SAMtools (v1.3.1), and transcript assembly was performed with StringTie (v1.3.6). Gene expression levels was assessed on the basis of TPM (transcripts per million) values for each mRNA. DEGs were identified from read count data using the DESeq2 software package with significance thresholds set at a false discovery rate (FDR) of <0.05 and |log_2_ fold change| > 1.

### Single-cell transcriptome analysis

*P. vlangalii* liver tissue was extracted and sent to BGI (Wuhan, China). Next-generation sequencing was produced through 10x Genomics. The filtered unique molecular identifier (UMI) count matrix was imported into R for downstream analysis. Cells with a mitochondrial RNA UMI greater than 15%, UMI totals below 200, and UMI totals greater than 7500 were removed to obtain high-quality data for all species. Variable features were identified with Seurat5 before scaling the data and running principal components analysis (PCA). Gene counts were normalized using the NormalizeData method, and 2000 highly variable genes were selected for downstream analysis. We used the typical CCA method for dataset integration to correct for batch effects. The union of the top 2000 genes with the highest dispersion in each dataset was taken, and anchors were identified using the FindIntegrationAnchors function. We then used the IntegrateData function to generate the integration expression matrix. We scaled the data and performed dimensionality reduction using the RunPCA function. Cells were clustered using the standard Seurat workflow and visualized using uniform manifold approximation and projection (UMAP).

### Indirect calorimetry

O_2_ and CO_2_ of mice were analyzed by the Panlab Oxylet System (Allentown Caging Equipment, USA), and lizard oxygen was analyzed by an automated flow-through respirometry system (TSE Systems, Bad Homburg, Germany) (*59*). *GCG*-KO mice, *P. vlangalii*, and *E. argus* were acclimatized in a metabolic chamber for 24 hours. Data were collected for each cage every 3 min for 24 hours at the same time for mice. Oxygen consumption rate (*V*O_2_, ml/hour) and carbon dioxide production rate (*V*CO_2_, ml/hour) for mice were calculated by the Metabolism software using the reference cages as (adjusted) measurements of oxygen and carbon dioxide inflow and were used to calculate *RER* (*RER* = *V*CO_2_/*V*O_2_) and *EE* = (3.815 + 1.232 × *RQ*) × *V*O_2_ × 1.44 (*RER* is the respiratory exchange ratio, *EE* is the energy expenditure, and *RQ* is the respiratory quotient). For lizards, the oxygen consumption rate (*V*O_2_, ml/hour) was calculated by the TSE software using the reference cages as (adjusted) measurements of oxygen inflow and analyzed using standard software (TSE Systems, Germany). The *V*O_2_ was calculated on the basis of the lowest rate of oxygen consumption over 5 min. Samples were collected during light and dark cycles and adjusted for body mass.

### Molecular docking prediction

The GCGR protein structure of *P. vlangalii* was predicted using Alpha Fold 3, and the three-dimensional (3D) structure of Ado was downloaded from PubChem (https://pubchem.ncbi.nlm.nih.gov/compound/91933867). To predict the binding affinity and interaction mode between the target compound Ado and the GCGR, semiflexible molecular docking was performed using AutoDock Vina integrated within UCSF Chimera (v1.17.3). Before docking, the receptor and ligand structures were preprocessed by adding polar hydrogens and assigning Gasteiger charges. A grid box with dimensions of 25 Å by 25 Å by 25 Å was established, centered at *X* = 15, *Y* = −10, and *Z* = 10, to encompass the anticipated binding pocket of GCGR. The exhaustiveness parameter was set to the default value of 8 to achieve an optimal balance between search space efficiency and accuracy, generating a maximum of 10 binding modes per ligand. The optimal binding conformation was selected on the basis of two primary criteria: the lowest docking score (binding energy, kcal/mol) and the formation of biologically favorable interactions with key residues within the GCGR binding site. The selected optimal conformation for Ado yielded a docking score of −7.11 kcal/mol.

For the docking between the peptide ligand Des and GCGR, a target-specific strategy was used. The 3D structure of the Des peptide (sequence: SQGTFTSEYSKYLDSRRAQDFVQWLMNT) was first modeled de novo using the PEP-FOLD3 server via the RPBS web Portal (http://bioserv.rpbs.univ-paris-diderot.fr/services/PEP-FOLD3/), and the top-ranked model (Model 1) was selected as the optimal conformation. Subsequently, virtual docking with GCGR was conducted using the HPEPDOCK 2.0 server (http://huanglab.phys.hust.edu.cn/hpepdock/). To accurately capture the dynamic binding conformation, the docking was guided by specifying key receptor binding residues (residues 134, 138, and 198). The optimal docking model was selected for visualization, yielding a final docking score of −253.52 (HPEPDOCK energy unit).

The two optimal docked complexes were further analyzed and visualized using PyMOL, which was used to generate both the overall 3D structural representations and detailed views of the specific interactions at the binding interfaces.

### Statistical analysis

Statistical analyses were performed using GraphPad Prism 8. Data represent means (± SD) from at least three independent experiments; each performed with three technical replicates. Comparisons between two groups were made using two-tailed unpaired Student’s *t* tests. A one-way analysis of variance (ANOVA) followed Tukey’s post hoc test was used for comparisons between the three groups. For dose-response experiments, data were normalized and analyzed using nonlinear curve fitting of log (ligand) versus response (four parameters) curves. Exact values of *n* (number of biological or experimental replicates) are shown in the figure legends, and *P* values are indicated in the graphical data. Statistical significance is defined as follows: n.s., not significant; *P < 0.05, **P < 0.01, ***P < 0.001, and ****P < 0.0001.

## Supplementary Materials

### The PDF file includes:

Figs. S1 to S6 Tables S1 to S3, S5 and S6, and S11 Legends for tables S4, S7 to S10, and S12 Legend for movie S1
