# Supplementary material for: Constitutively active glucagon receptor (GCGR) physiologically regulates body temperature in lizards
Source: Sci Adv. 2026 Aug 21;12(34):eaeb8744. doi: 10.1126/sciadv.aeb8744 (PMC13496201; doi:10.1126/sciadv.aeb8744)
Supplement: Supplementary file 1 [file sciadv.aeb8744_sm.pdf]

Science Advances
AAAS

Supplementary Materials for

Constitutively active glucagon receptor (GCGR) physiologically regulates body temperature in lizards

Songsong Liu *et al.*

Corresponding author: Cheng Deng, dengcheng@wchscu.cn

Sci. Adv. **12**, eaeb8744 (2026)
DOI: 10.1126/sciadv.aeb8744

The PDF file includes:

Figs. S1 to S6
Tables S1 to S3, S5 and S6, and S11
Legends for tables S4, S7 to S10, and S12
Legend for movie S1

Other Supplementary Material for this manuscript includes the following:

Tables S4, S7 to S10, and S12
Movie S1

Supplementary Figures

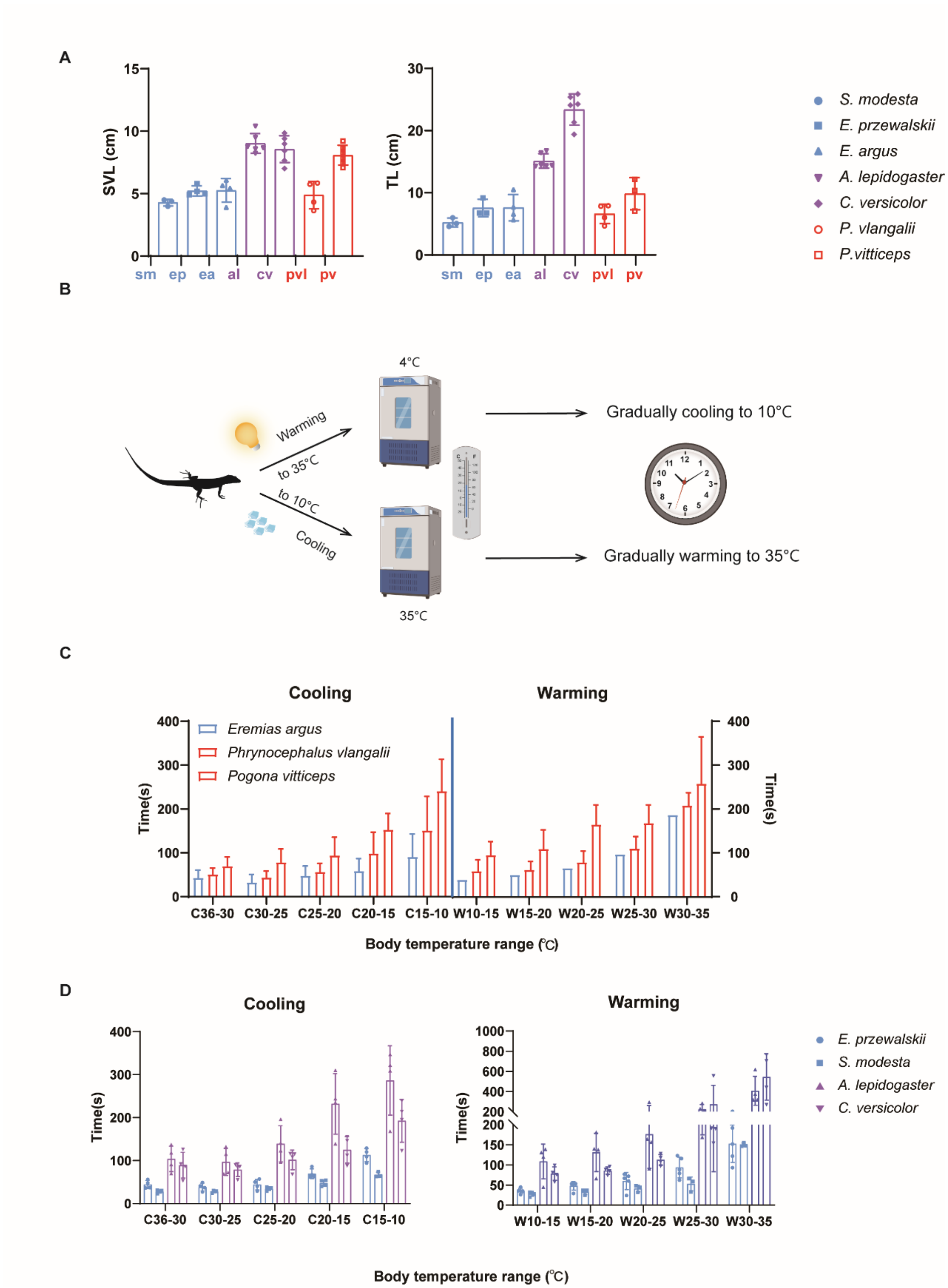

Supplementary Figure 1. Thermal reaction constants in different lizards show their abilities to regulate temperature in both directions. A. Total length (TL) (n=3-6) and snout-vent length (SVL) (n=3-6) of different lizard species-these indicators are associated with somatic maturity. **B.** Lizards were warmed under a lamp until they reached 35°C. Subsequently they were

placed in an incubator at 4℃ while temperature was monitored and the time it took to reach each 5℃ interval (cloacal temperature) was noted. In a second experiment, lizards were chilled on ice to 4℃ and then placed in a 37℃ incubator to monitor the times it took to warm up, starting the timer when the lizards reached 5°C (cloacal temperature), noting the times it takes to reach each 5℃ interval up to 35℃. **C.** Time intervals *E. argus*, *P. vlangalii* and *P. vitticeps* required for each 5℃ step for cooling (left) and warming (right) (n=3-4). **D.** Time intervals time of *E. przewalskii*, *S. modesta*, A. *Lepidogaster* and *C. versicolor* to lower (C) or raise (W) temperature at 5℃ intervals (n=3-4).

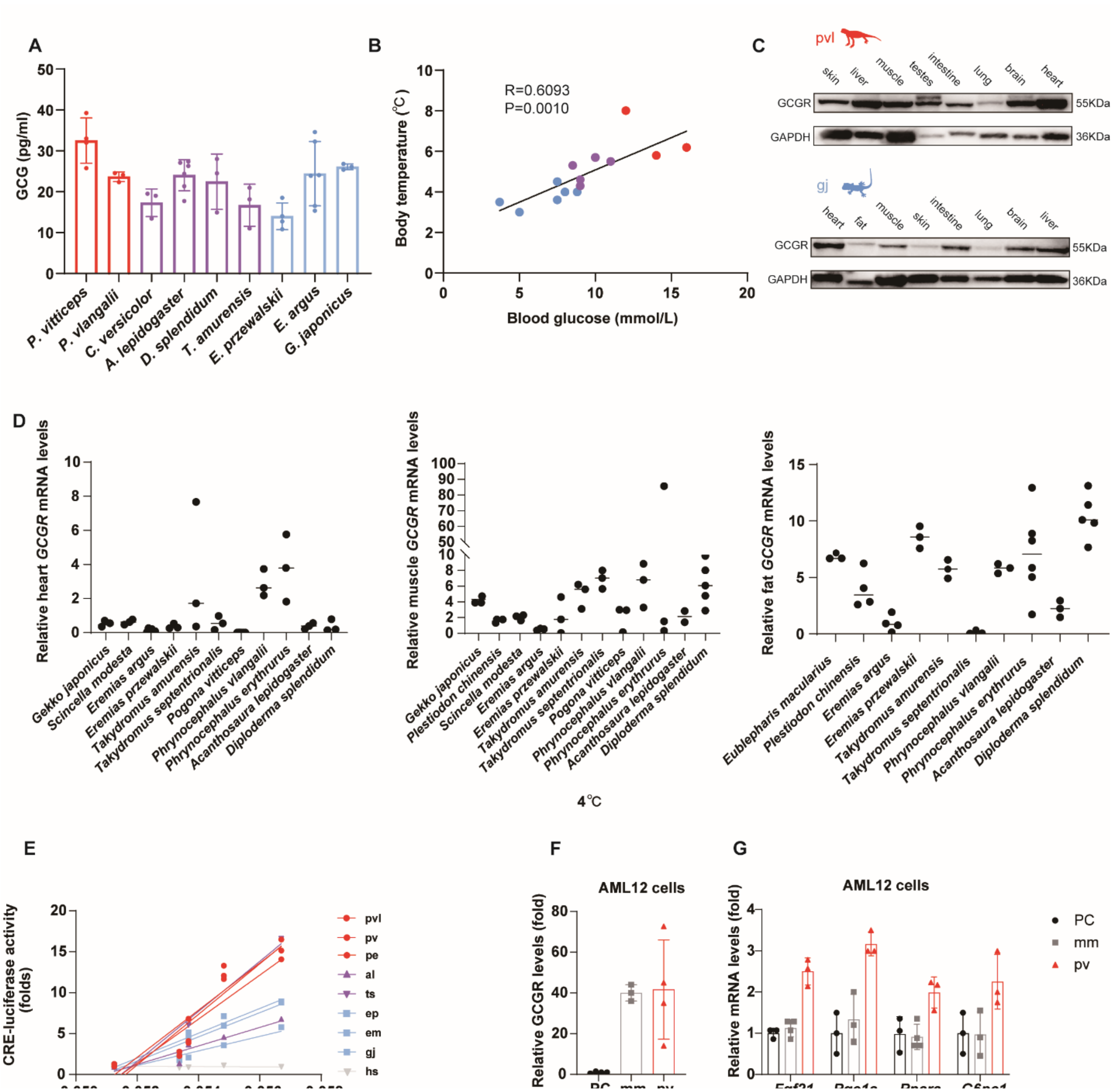

Supplementary Figure 2. Transfection of lizard *GCGR* in hepatocytes results in changes in cell-associated thermogenic genes. A-D, related to Fig. 1. In the same experimental setup as in Fig. 1a we measured **A.** Concentration of GCG in different lizard species after the third cold exposure (n=3-5). **B.** Linear correlation of body temperature and blood glucose of 14 different lizard species. Species from left to right: em, gj, pc, sm, ea, ep, ta, ts, cv, al, ds, pv, pvl, pe. **C.** GCGR protein expression in heart, muscle, and fat of *P. vlangalii* (above, n=1) and *G. japonicus* (bottom, n=1) by Western blotting. **D.** *GCGR* mRNA expression in heart, muscle and fat of various lizard species (n=3-6). **E.** GCGR constitutive activity of various lizards. **F,G** AML12 mouse liver cells transfected with empty pcDNA3.1 vector or those containing V5-tagged mouse mm*GCGR*, orV5-tagged pv*GCGR* gene, measuring exogenous *GCGR* mRNA levels 48 hours after transfection using qRT-PCR (n=3-4) **(F)** and endogenous mRNAs encoded by genes related to thermogenesis and glycogenolysis (*Fgf21, Pgc1a, Ppara* and *G6pc1*) (n=3-4) **(G)**.

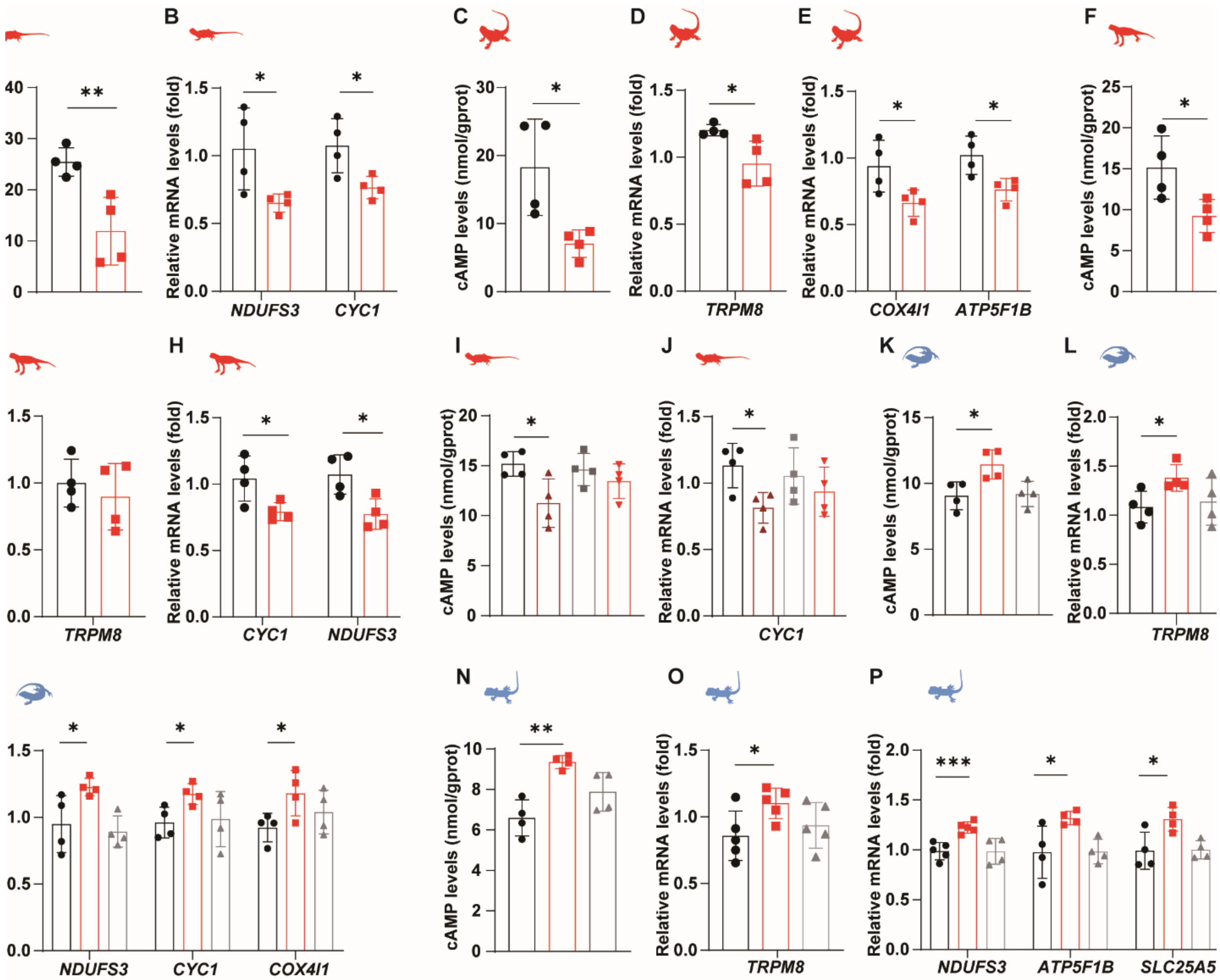

Supplementary Figure 3. cAMP levels and expression of mitochondria-related thermogenic genes across lizard species. A–P, related to Fig. 1–4. After the third two hour exposure of animals to 4°C we measured **A.** Hepatic cAMP levels (n=4) of *P. vlangalii*. **B.** Hepatic mRNA expression levels of genes associated with mitochondrial oxidative phosphorylation (*NDUFS3*, *CYC1*) of *P. vlangalii*. **C.** Hepatic cAMP levels (n=4) of *P. vitticeps*. **D.** Hepatic mRNA expression levels of genes associated with cold sensation (*TRPM8*) of *P. vitticeps* (n=4). **E.** Hepatic mRNA expression levels of genes involved in mitochondrial oxidative phosphorylation (*COX4I1*, *ATP5F1B*) of *P. vitticeps* (n=4). **F.** Hepatic cAMP levels (n=4) of *P. erythrurus*. **G.** Hepatic mRNA expression levels of genes associated with cold sensation (*TRPM8*) of *P. erythrurus* (n=4). **H.** Hepatic mRNA expression levels of genes involved in mitochondrial oxidative phosphorylation (*CYC1*, *NDUFS3*) of *P. erythrurus* (n=4). After intrahepatic injection with Ado and Des, *P. vlangalii* was directly exposed to 4°C for two hours and then we measured **I.** Hepatic cAMP levels (n=4) of *P. vlangalii*. **J.** Hepatic mRNA expression levels of genes associated with mitochondrial oxidative phosphorylation (*CYC1*) of *P. vlangalii* (n=4). After the third two hour exposure to 4°C we measured **K.** Hepatic cAMP levels (n=4) of *E. argus*. **L.** Hepatic mRNA expression levels of genes associated with cold sensation (*TRPM8*) of *E. argus* (n=4). **M.** Hepatic mRNA expression levels of genes associated with mitochondrial oxidative phosphorylation (*NDUFS3*, *CYC1*, *COX4I1*) of *E. argus* (n=4). **N.** Hepatic cAMP levels (n=4) of *G. japonicus*. **O.** Hepatic mRNA expression levels of genes associated with cold sensation

(*TRPM8*) of *G. japonicus* (n=4). **P.** Hepatic mRNA expression levels of genes associated with mitochondrial oxidative phosphorylation (*NDUFS3*, *ATP5F1B*) and mitochondrial energy transport (*SLC25A5*) of *G. japonicus* (n=4).

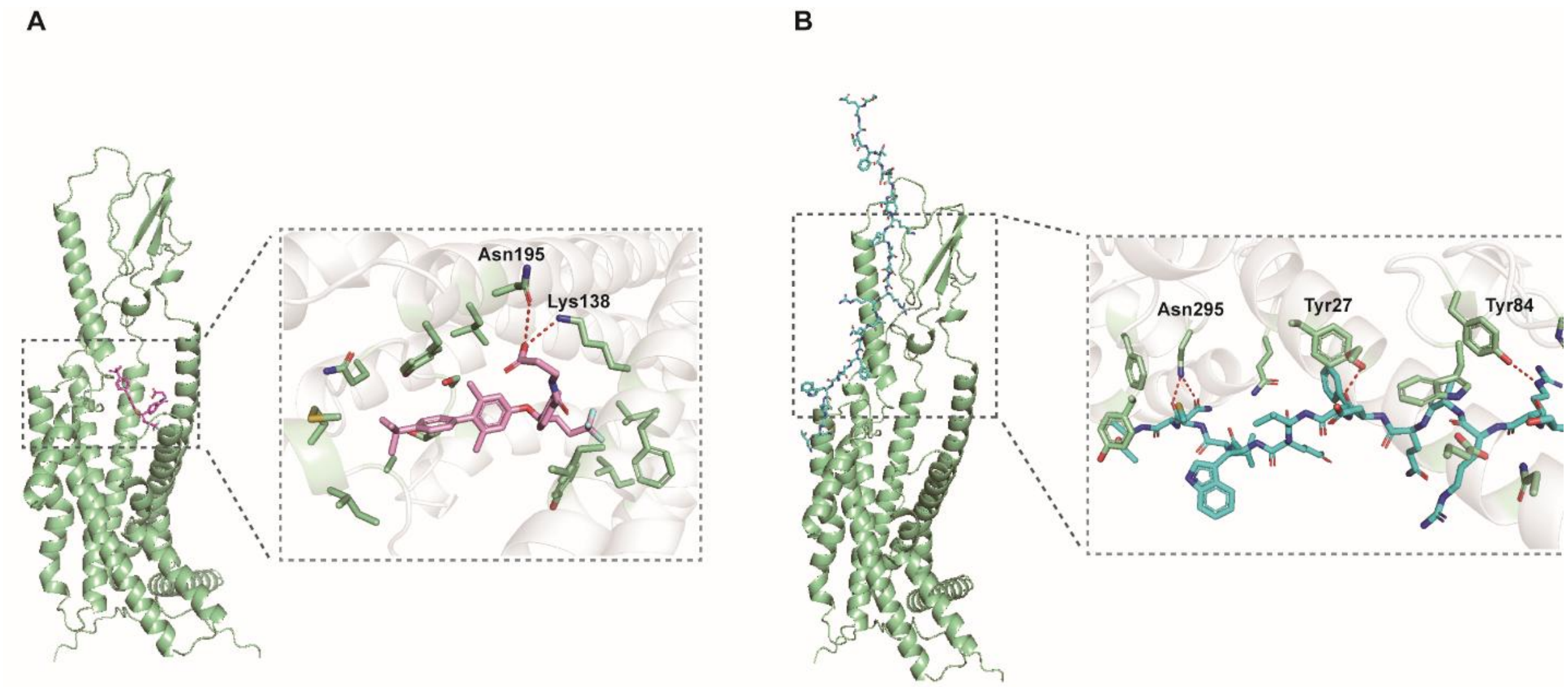

Supplementary Figure 4. Molecular docking of GCGR inhibitors with *P. vlangalii* GCGR. 3D PyMOL visualizations of the molecular docking results showing both global conformations and magnified local binding modes. **A.** Interactions between Ado (pink) and GCGR of *P. vlangalii*. **B.** Interactions between Des (cyan) and GCGR of *P. vlangali*i. In the magnified views, the interacting receptor amino acid residues are colored in pale green. Hydrogen-bonding interactions are indicated by red dashed lines, and the specific interacting residues are labeled.

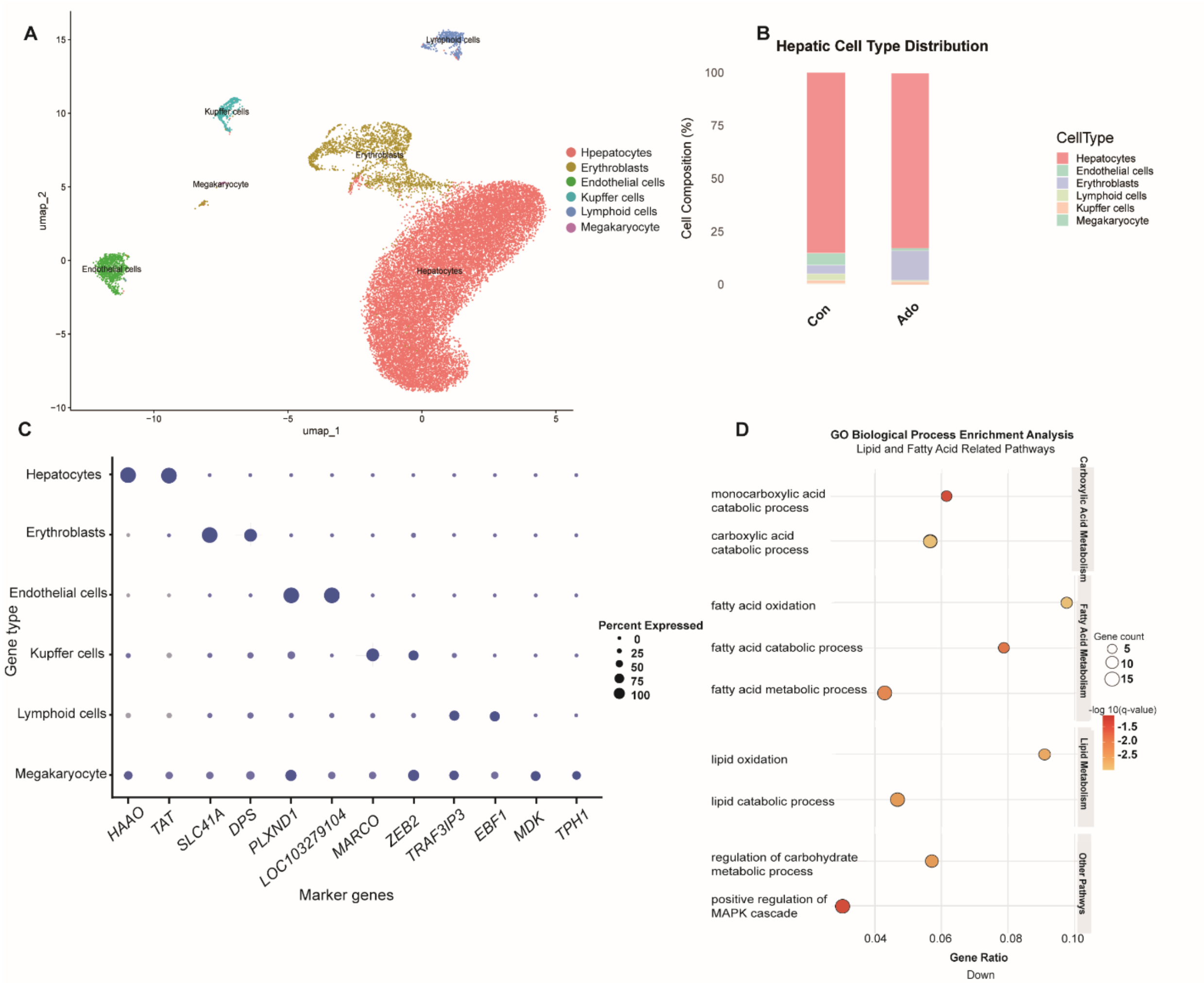

Supplementary Figure 5. Liver single-cell nuclear analysis suggests that constitutively active GCGR causes elevation of thermogenesis-related genes in lizards at low temperatures. A-D. Single nucleus RNA-seq analysis of *P. vlangalii* liver two hours after injection with control and adomeglivant (Ado) group at 4°C (n=1). Integration of sequenced *P. vlangalii* cells in liver and stratification into six different cell types **(B)** and Uniform Manifold Approximation and Projection (UMAP) plots showing different cell types from single nuclei RNA data from *P. vlangalii* **(A)**. Top 2 marker genes for each cell type **(C)**. KEGG pathways enriched for down-regulated genes by differential gene expression analysis. The KEGG pathways related to fatty acid metabolism, and carbon metabolism **(D)**.

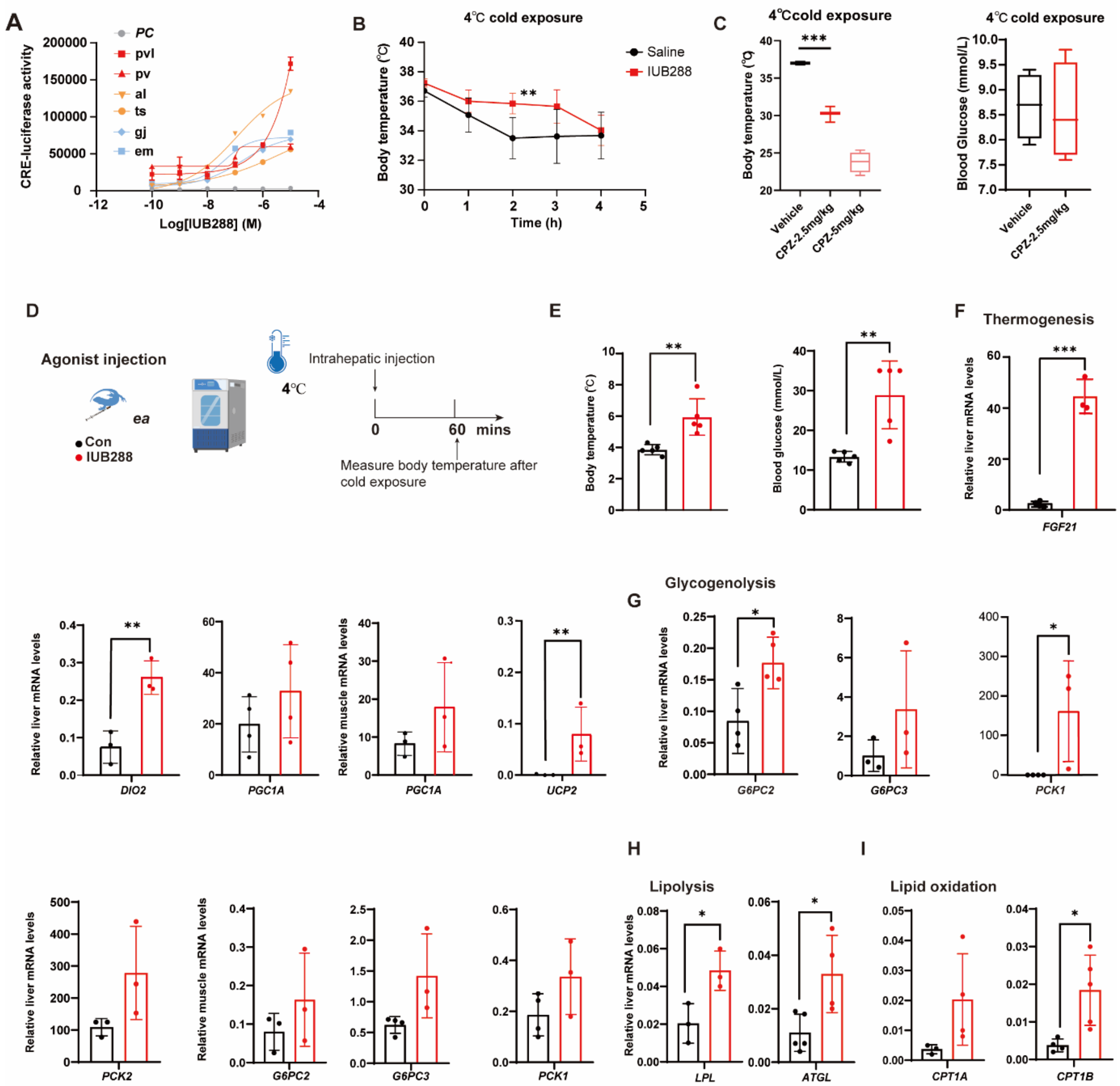

Supplementary Figure 6. GCGR agonist can also facilitate warming in hypothermic lizards. GCGR of *P. vlangalii* and *P. vitticeps* can be further stimulated by the GCGR agonist IUB288 as detected by luciferase assay in HEK293T cells **(A)**. Male ICR mice (6-8 weeks) received intraperitoneally (i.p.) saline or IUB288 (10 nmol/kg). Body temperature was measured after exposure to 4℃ for indicated time points (n=6) **(B)**. Male ICR mice (6-8 weeks) received intraperitoneally (i.p.) vehicle or CPZ (2.5mg/kg or 5mg/kg). Body temperature (left) and blood glucose (right) were measured after exposure to 4℃ for one hour (n=3-4) **(C)**. Red represents CPZ-2.5mg/kg group; pink represents CPZ-5mg/kg group and black represents vehicle group. Intrahepatic injection of the GCGR agonist IUB288 into the liver of *E. argus* **(D)**, and the body temperature (left, n=5) and blood glucose (right, n=5) **(E)** were measured after cold exposure at 4℃ for 60 minutes after injection. After *E. argus* was injected with IUB288, the expressions of thermogenesis-related genes (n=3-4) **(F)**, glycogenolysis-related genes (n=3-4) **(G)**, lipolysis genes (n=3) (**H**), and lipid oxidation genes (n=3-4) (**I**) in the liver were upregulated. Red represents IUB288 group; black represents control (Con) group.

Supplementary Tables.

Supplementary Table S1. Abbreviations for genes and other proper nouns used in this study.

Abbreviation	Full title
AAV	Adeno-associated virus
ACACA	Acetyl-CoA Carboxylase
Ado	Adomeglivant
AML12	Alpha Mouse Liver 12 cell line
ATGL	Adipose triglyceride lipase
ATP5F1B	ATP Synthase F1 Subunit Beta
COX4I1	Cytochrome C Oxidase Subunit 4I1
CPT1A	Carnitine palmitoyltransferase 1A
CPT1B	Carnitine palmitoyltransferase 1B
CYC1	Cytochrome C1
Des	DesHis1Pro4-glucagon amide
DMEM	Modified Eagle Medium
DIO2	Iodothyronine Deiodinase 2
FASN	Fatty acid synthase
FGF21	Fibroblast growth factor 21
GCG	Glucagon receptor
GCGR	Glucagon receptor
GPCRs	G protein-coupled receptors
G6PC1	Glucose-6-phosphatase 1
G6PC2	Glucose-6-phosphatase 2
G6PC3	Glucose-6-phosphatase 3
HEK293T	Human Embryonic Kidney 293T cells
KEGG	Kyoto Encyclopedia of Genes and Genomes
LPL	Lipoprotein lipase
NDUFS3	NADH dehydrogenase [ubiquinone] iron-sulfur protein 3
PEPCK1 (PCK1)	Phosphoenolpyruvate carboxykinase 1
PEPCK2 (PCK2)	Phosphoenolpyruvate carboxykinase 2
PGC1A	Peroxisome Proliferator-Activated Receptor Gamma, Coactivator 1 Alpha
PKA	Protein Kinase A
PPARA	Peroxisome proliferator-activated receptor alpha
PPARG	Peroxisome proliferator-activated receptor gamma
RQ	Respiratory quotient
SIRT1	Sirtuin 1
SLC25A5	Solute Carrier Family 25 Member 5
sNuc-RNAseq	Single-nucleus RNA-sequencing
TRPM8	Transient Receptor Potential Cation Channel Subfamily M Member 8
UCP2	Uncoupling Protein 2
UCP3	Uncoupling Protein 3
UMAP	Uniform manifold approximation and projection
VCO2	Carbon dioxide production
VO2	Oxygen consumption

Supplementary Table S2. Abbreviations for species used in this study.

Species	Latin name	Common name
al	*Acanthosaura lepidogaster*	Brown Pricklenape
ds	*Diploderma splendidum*	Splendid japalure
cv	*Calotes versicolor*	Oriental Garden Lizard
ea	*Eremias argus*	Mongolian race runner
ep	*Eremias przewalskii*	Gobi race runner
em	*Eublepharis macularius*	Leopard Gecko
gj	*Gekko japonicus*	Yellow-Striped Skink
pvl	*Phrynocephalus vlangalii*	Vlangals toad-headed agama
pe	*Phrynocephalus erythrurus*	Red-tailed toad-headed agama
pv	*Pogona vitticeps*	Bearded dragon
pc	*Plestiodon chinensis*	Chinese skink
sm	*Scincella modesta*	Ningbo smooth skink
ta	*Takydromus amurensis*	China grass lizard
ts	*Takydromus septentrionalis*	Amur Grass Lizard

Supplementary Table S3. Average time schedules for experiments on thermal reaction constants.

Latin name	Latin abbreviation	(τw)	(τc)	τw/τc	g
Pogona vitticeps	pv	3.04136253	4.750593824	0.6402068	13.77
Scincella modesta	sm	2.747252747	3.721622627	0.7381868	1.13
Eremias przewalskii	ep	3.347840643	3.944773176	0.8486776	2.98
Acanthosaura lepidogaster	al	5.473453749	5.714285714	0.9578544	21.28
Phrynocephalus vlangalii	pvl	2.927400468	2.631578947	1.1124122	4.58
Eremias argus	ea	2.787068004	1.563721658	1.78233	2.9
Calotes versicolor	cv	16.36661211	3.144654088	5.2045827	16.4

Supplementary Table S4. Information for primers used in this study.

Supplementary Table S5. Source of information on the antibodies used in this study.

Antibody name	Supplier name	Catalog number	Object
GCGR	General gene	UPA61074	Western blot
beta Actin Ab (HRP)	Abways	AB2001	Western blot
V5-tag	Cell Signaling Technnology	13202S	Western blot
FGF2121	Abways	Q9NSA1	Western blot
p-AKT (S473) D9E	CST	4060S	Western blot
AKT (pan) C67E7	CST	4691S	Western blot

Supplementary Table S6. AAV vector name and serotype.

Adeno-Associated Virus	Serotype
pAAV-CAG-MCS-3xFLAG-WPRE	2/8
pAAV-CAG-central bearded dragon-gcgr-V5-WPRE	2/8
PAAV-U6-ShRNA(NC)-CMV-EGFP-WPRE	2/8
PAAV-U6-ShRNA(GCGR)-CMV-EGFP-WPRE	2/8

Supplementary Table S7. Cooling times for lizards**.**

Supplementary Table S8. Warming times for lizards**.**

Supplementary Table S9. DEGs for ICR mice**.**

Supplementary Table S10. Pathways enriched by the two sets of differentially expressed genes identified through single-nucleus RNA sequencing (snRNA-seq) analysis of lizard liver.

Supplementary Table S11. Information on all software and equipment used in this study.

software	version	object
ImageJ	v1.54g	Fluorescence microscope image, Western blot analysis
Graph pad	v10.2.0	Data analysis
EnVision Manager	1.14.3049.1193	Chemiluminescence detection
QuantStudioTM Design & Analysis Software	v1.5.2	RT-PCR
NIS-Elements BR	v5.21	Fluorescence microscope
Maga	v10.2.6	Phylogenetic tree analysis
Image labTM Touch Software	v2.4.0.03-DEV MODE	Western blot imaging
TanonImage	v1.00	Nucleic acid electrophoresis
Metabolism	v3.0	Respiratory metabolism (mice)
TSE LabMaster	v5.2.4	Respiratory metabolism (reptiles)

Supplementary Table S12. GCGR plasmid information used in this study.

Supplementary Movie

Supplementary Movie S1. In situ injection of liver into lizards.
